# Age-related molecular IgE sensitization to indoor and outdoor respiratory allergens: a two-step cross-sectional study in Algeria and Türkiye

**DOI:** 10.1016/j.ebiom.2026.106484

**Published:** 2026-09-24

**Authors:** Lydia Lamara Mahammed, Susanne Vrtala, Oluwatoyin Akinfenwa, Maria-Roxana Buzan, Kuan-Wei Chen, Alp Kazancioglu, Raffaela Campana, Monica-Daniela Cotarcă, Mirela Curin, Yulia Dorofeeva, Nishelle Dsouza, Alexandra Dubovets, Margarete Focke-Tejkl, Pia Gattinger, Heinrich Grausgruber, Huey-Jy Huang, Alexander Karaulov, Evgenii Kozlov, Andrea Krisai, Lun Li, Birgit Linhart, Carmen Panaitescu, Eszter Sarzsinszky, Daria Trifonova, Mikhail Tulaev, Milena Weber, Brahim Belaid, Recep Sancak, Mehmet Halil Celiksoy, Lynda Sekfali, Nafissa Benhalla, Merzak Gharnaout, Bulent Enis Sekerel, Rudolf Valenta, Reda Djidjik

**Affiliations:** a Department of Medical Immunology, Beni-Messous Teaching Hospital, Algiers, Algeria; b Faculty of Pharmacy, University of Health Sciences, Algiers, Algeria; c Department of Pathophysiology and Allergy Research, Center for Pathophysiology, Infectiology and Immunology, Medical University of Vienna, Vienna, Austria; d Department of Functional Sciences, Center of Immuno-Physiology and Biotechnologies, Victor Babes University of Medicine and Pharmacy, Timisoara, Romania; e OncoGen Center, Pius Brinzeu County Clinical Emergency Hospital, Timisoara, Romania; f Department of Pediatric Allergy and Immunology, Faculty of Medicine, Hacettepe University, Ankara, Türkiye; g Laboratory of Immunopathology, Department of Clinical Immunology and Allergy, Sechenov First Moscow State Medical University, Moscow, Russia; h Life Improvement by Future Technologies (LIFT) Center, Moscow, Russia; i Department of Agricultural Sciences, Institute of Crop Breeding and Genomics, BOKU University, Tulln an der Donau, Austria; j Center for Molecular Allergology, Karl Landsteiner University of Health Sciences, Krems, Austria; k Department of Allergy, Peking Union Medical College Hospital, Chinese Academy of Medical Sciences, Peking Union Medical College, Beijing, China; l Department of Pediatric Allergy and Immunology, Ondokuz Mayis University, Samsun, Türkiye; m Department of Pediatric Allergy and Immunology, Başakşehir Çam and Sakura City Hospital, University of Health Sciences, Istanbul, Türkiye; n Department of Pediatrics, Beni-Messous Teaching Hospital, Algiers, Algeria; o Faculty of Medicine, University of Health Sciences, Algiers, Algeria; p Department of Pulmonology and Allergy, Beni-Messous Teaching Hospital, Algiers, Algeria

**Keywords:** Allergy, Allergen, Exposome, Population study, Allergen-micro-array

## Abstract

**Background:**

Immunoglobulin E-mediated allergy is the most important hypersensitivity disease affecting more than 30% of the population worldwide. The analysis of molecular IgE sensitization profiles in defined populations and age groups is important for the development of allergen-specific preventive and therapeutic interventions. The present study was performed to examine if there are differences regarding molecular IgE reactivity profiles in children and adults from two regions with similar climate and exposome.

**Methods:**

This two-step cross-sectional study assessed molecular IgE reactivity profiles using an experimental allergen microarray comprising 105 allergen molecules. In the first step, an exploratory cross-sectional study, serum samples from 539 allergic patients (259 children, 280 adults) who were born and grew up in the Algiers region were analyzed. The second step was a prospective cross-sectional study performed in 272 Turkish allergic patients (133 children, 139 adults) from Istanbul and Samsun regions with a climate and allergen exposome similar to Algiers, with the goal of investigating the reproducibility of possible differences in molecular IgE sensitization profiles between children and adults.

**Findings:**

In both the exploratory Algiers study and the prospective Turkish study, significantly more children than adults with respiratory allergy were sensitized to major respiratory house dust mite allergens (Algiers: Der p 1, Der p 2: *p* < 0.001 and Der p 23: *p* = 0.001; Türkiye: Der p 1: *p* = 0.018 and Der p 2: *p* = 0.019) whereas significantly more adults than children were sensitized to regionally common respiratory outdoor allergens (Algiers: Ole e 1: *p* = 0.037; Phl p 1: *p* = 0.046; Türkiye: Ole e 1: *p* = 0.016; Amb a 1: *p* = 0.002; Amb a 8: *p* < 0.001). Asthma was significantly more common in children than in adult patients with respiratory allergy (Algiers: *p* < 0.001; Türkiye: *p* = 0.034).

**Interpretation:**

Our study demonstrates different molecular IgE sensitization profiles in two age groups (children and adults) on two different continents with a similar climate and allergen exposome which may be due to exogenous factors such as climate change, different lifestyles, pollution and changes in vegetation. Both major house dust mite sensitization and asthma were more common in children, suggesting sensitization profile may contribute to asthma risk.

**Funding:**

Danube Allergy Research Cluster, Country of Lower Austria, Medical University of Vienna and Russian Science Foundation.

**Research in context**

**Evidence before this study**

Immunoglobulin E-mediated allergy is the most common hypersensitivity disease affecting more than one third of the population. Prior research has established that IgE sensitization is affected by multiple endogenous and exogenous factors. Among exogenous factors, early-life contact with allergens from the environment (i.e., allergen exposome) determines the allergen sensitization profile of an allergic person. In addition, environmental factors such as hygienic conditions at birth (e.g., cesarean section) and early life (e.g., rural *vs* urban environment, anthroposophic lifestyle) were reported to influence the general frequency of IgE sensitization. The possibility of investigating molecular IgE sensitization profiles with comprehensive panels of micro-arrayed allergen molecules has enabled the study of the evolution of molecular IgE sensitization profiles in birth cohorts and confirmed that the early-life allergen exposome determines life-long allergen sensitization profiles. However, detailed investigations into differences in respiratory-allergen sensitization profiles between age groups, such as children and adults from the same region, and their associations with allergic symptoms are lacking.

**Added value of this study**

This study is a two-step study comprising an exploratory cross-sectional analysis of molecular IgE sensitization profiles in the Algiers region and a prospective cross-sectional study performed in a region with similar climate and allergen exposome in Türkiye. In both steps of the study, significantly more children than adults with respiratory allergy were sensitized to major respiratory house dust mite allergens whereas significantly more adults than children were sensitized to regionally common respiratory outdoor allergens. Asthma was significantly more common in children than in adult patients with respiratory allergy.

**Implications of all the available evidence**

The present findings highlight that molecular sensitization patterns may differ substantially between age groups within regions with comparable climate and allergen exposome, suggesting a potential role of environmental and lifestyle-related factors in shaping sensitization profiles. Further prospective longitudinal studies investigating the impact of environmental and lifestyle-related factors on molecular IgE sensitization profiles and allergic phenotypes are warranted. Whether major house dust mite sensitization directly contributes to asthma risk, independent of age, is a link future studies should test directly, adjusting for age.

## Introduction

The development of component-resolved diagnostics (CRD),^1^ also known as molecular diagnosis, represents a step forward in the diagnosis of allergic diseases based on purified and/or natural allergens.^2^ Molecular allergy diagnosis allows a precise identification of the genuinely sensitizing allergen sources that can be obscured by cross-reactivity when allergen extracts are used. Molecular diagnosis is increasingly integrated into clinical practice to characterize complex allergic phenotypes,^3^ to stratify disease severity, and to assess the risk of severe allergic reactions.^4^ Molecular allergy diagnosis also plays a critical role in guiding allergen-specific therapeutic strategies, including allergen avoidance measures and the accurate prescription and monitoring of allergen immunotherapy (AIT).^5^

Molecular allergy diagnosis was advanced by the introduction of the microarray technology in 2002,^6^ which allows simultaneous IgE antibody testing of multiple allergens with a tiny amount of serum. The microarray-based allergy diagnosis has particularly influenced the field of research by addressing several fundamental research questions and providing detailed information on the individual sensitization profile in large-scale studies of different populations. Notably, it offers the opportunity to analyze the evolution of IgE reactivity profiles over time in longitudinal birth cohorts. In this context, a study was conducted by Hatzler et al.^7^ in 2012, who demonstrated that IgE responses to timothy grass during childhood may increase in molecular complexity over time through a “molecular spreading” process, with Phl p 1 identified as the initial sensitizing allergen in more than 75% of patients. Similarly, further studies^8^, ^9^, ^10^, ^11^, ^12^, ^13^, ^14^, ^15^, ^16^ have investigated the evolution of molecular IgE sensitization profiles to allergens from various allergenic sources (e.g., HDM, cat, dog) and have demonstrated that early assessment of IgE sensitization profiles and IgE levels allows predicting the course and severity of allergic diseases later in life.^8^^,^^13^ Thus early childhood is the cradle of allergic sensitizations whereas de novo sensitizations are rare events in allergic adults.^17^

There is evidence that allergen sensitization profiles could be influenced by environmental and geographic determinants. For example, it was observed that changes in vegetation occurring through coordinated programs and/or natural changes can alter the allergic sensitization profiles towards plant-derived allergens in populations within two generations.^18^ In another study published in 2023, it was found that sensitization profiles to allergen molecules strongly depend on the regional allergen exposome and climate in the different cohorts from Europe.^19^

All these data highlight the importance of population-based studies to promote better understanding of allergen sensitization patterns. Presently, such data are available only for few regions, mainly in Europe. There are huge gaps in knowledge regarding Asia, America and Africa, especially from North Africa. Existing data are limited to assessments of sensitization to a few selected molecular allergens in selected patients,^20^, ^21^, ^22^, ^23^, ^24^ and one study conducted in the context of primary immune-deficiencies.^25^ In Algeria, the largest country in Africa, some regional studies have described the sensitization profile of allergic patients mainly to allergenic sources, indicating the predominance of house dust mite (HDM) sensitization for respiratory allergens.^26^^,^^27^ The aim of this study was to examine if there are differences regarding molecular IgE reactivity profiles in children and adults in two countries from two continents with similar climate and allergen exposome. This question has clinical relevance because it has been shown that certain molecular IgE sensitization profiles are associated with phenotypes and symptoms of allergic diseases.^3^^,^^8^^,^^10^^,^^11^^,^^13^ For this purpose, we conducted a two-step cross-sectional study. In the first exploratory cross-sectional study conducted in the Algiers region, we investigated whether there are differences in molecular IgE reactivity profiles to respiratory allergens in two age groups of patients with respiratory allergy (i.e., children *vs* adults) and if such differences may be associated with disease phenotypes, in particular asthma. We then conducted a second, prospective cross-sectional study in Türkiye, a region on another continent with a similar climate and allergen exposome, to test whether the same age-related differences in HDM and outdoor allergen sensitization, and the association between HDM sensitization and asthma, would also be observed in this independent population.

## Methods

### Study design and patient selection

Our study was a two-step cross-sectional allergen micro-array profiling study comprising first, an exploratory molecular IgE profiling analysis of previously collected sera from clinically characterized allergic adults and children (children: <18 years; adults: ≥18 years) from the Algiers region in North Africa (Fig. 1, Table 1) and second, a prospectively collected cross-sectional validation cohort designed to assess whether key findings made in children and adults of the exploratory first study can be also observed in allergic individuals (i.e., children: <18 years; and adults: ≥18 years) (Fig. 1, Table 2) from another region in another continent, namely two Turkish cities (i.e., Istanbul and Samsun) with comparable climatic and allergen-exposure characteristics. Sex was ascertained by clinicians who saw the subjects and the diagnosis of rhinitis, asthma and atopic dermatitis was performed by clinicians trained in allergy following international guidelines.^28^^,^^29^ Serum samples were stored at −20°C (Türkiye) or −80°C (Algiers) until analysis.

**Fig. 1:**
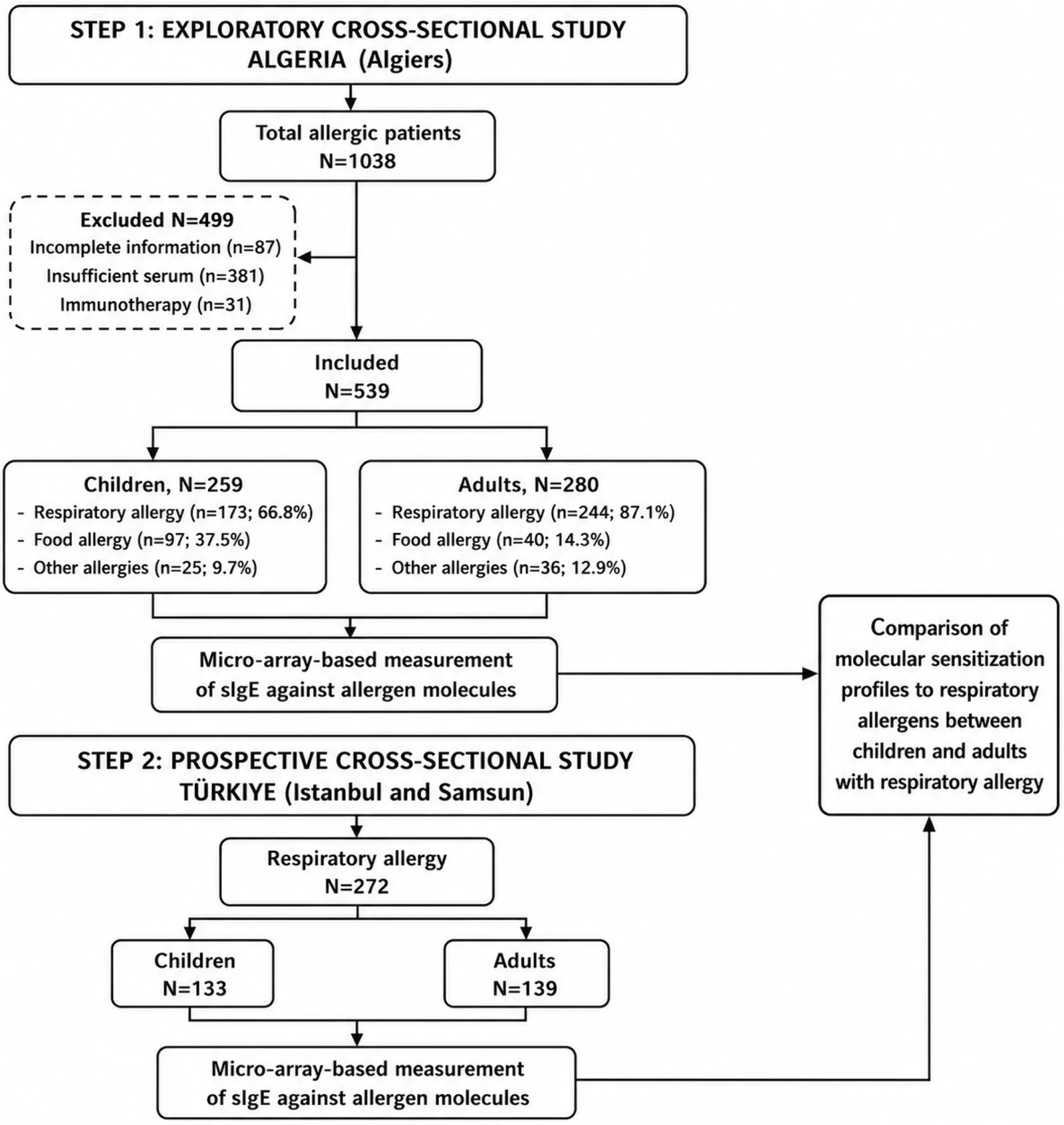
Flowchart of the study design and patient enrollment. The exploratory cohort included 539 patients from Algeria (Algiers) and the validation cohort included 272 patients from Türkiye (Istanbul and Samsun).

**Table 1:** Demographic and clinical characteristics of the Algerian (Algiers) study population (N = 539).

Characteristics	Children (N = 259)	Adults (N = 280)
Age, years		
Median (IQR)	8 (4–11)	32 (26–43)
Range	0.2–17	18–72
Sex, n/N (%)		
Male	164/259 (63.3)	123/280 (43.9)
Female	95/259 (36.7)	157/280 (56.1)
Clinical data, n/N (%)		
Respiratory allergy	173/259 (66.8)	244/280 (87.1)
Rhinitis	114/173 (65.9)	189/244 (77.5)
Asthma^a^	99/173 (57.2)	97/244 (39.8)
Conjunctivitis	28/173 (16.2)	41/244 (16.8)
Urticaria	28/173 (16.2)	40/244 (16.4)
Food allergy	97/259 (37.5)	40/280 (14.3)
Mucocutaneous symptoms	57/97 (58.8)	24/40 (60)
Anaphylaxis	22/97 (22.7)	12/40 (30)
Gastrointestinal symptoms	22/97 (22.7)	2/40 (5)
Oropharyngeal symptoms	4/97 (4.1)	6/40 (15)
Venom allergy	25/259 (9.7)	31/280 (11.1)
Latex allergy	0/259 (0)	5/280 (1.8)
Sensitization profile (allergen extracts), n/N (%)		
Respiratory allergy	173/259 (66.8)	244/280 (87.1)
House dust mites	160/173 (92.5)	199/244 (81.5)
Cat	56/173 (32.4)	59/244 (24.2)
Dog	21/173 (12.1)	26/244 (10.7)
Molds	4/173 (2.3)	10/244 (4.1)
Tree pollen	55/173 (31.8)	104/244 (42.6)
Grass pollen	18/173 (10.4)	50/244 (20.5)
Weed pollen	13/173 (7.5)	36/244 (14.8)
Food allergy	97/259 (37.5)	40/280 (14.3)
Milk	60/97 (61.9)	2/40 (5)
Shrimp	16/97 (16.5)	29/40 (72.5)
Legumes	17/97 (17.5)	4/40 (10)
Egg	15/97 (15.5)	1/40 (2.5)
Fish	9/97 (9.3)	2/40 (5)
Tree nuts	7/97 (7.2)	2/40 (5)
Fruits	6/97 (6.2)	2/40 (5)
Others	3/97 (3.1)	2/40 (5)
Venom allergy	25/259 (9.7)	31/280 (11.1)
Honeybee	24/25 (96)	29/31 (93.5)
Vespid (Yellow Jacket)	7/25 (28)	09/31 (29)
Area of living^b^, n/N (%)		
Urban	90/102 (88.2)	185/206 (89.8)
Rural	12/102 (11.8)	21/206 (10.2)
Coastal zone	100/102 (98)	198/206 (96)
High plateaus	2/102 (2)	6/206 (3)
Arid Zone (Saharan zone)	0/102 (0)	2/206 (1)

a Significantly more frequent in children than in adults (*p* < 0.001; χ^2^ test).

b Information obtained only from 308 patients with respiratory allergies (102 children and 206 adults).

**Table 2:** Demographic and clinical characteristics of the Turkish (Istanbul and Samsun) study population.

Characteristics	Children (N = 133)	Adults (N = 139)
Age, years		
Median (IQR)	7.7 (6.2–9.8)	32 (25–41)
Range	2.2–16.1	18–74
Sex, n (%)		
Male	83 (62.4)	60 (43.2)
Female	50 (37.6)	79 (56.8)
Clinical data, n (%)		
Allergic rhinitis	123 (92.5)	138 (99.3)
Asthma^a^	100 (75.2)	88 (63.3)
Atopic dermatitis	37 (27.8)	21 (15.1)
Food allergy	29 (21.8)	14 (10.1)
Sensitization profile (allergen extracts), n (%)		
House dust mites	113 (85)	107 (77)
Cat	17 (12.8)	31 (22.3)
Dog	10 (7.5)	21 (15.1)
Molds	12 (9)	1 (0.7)
Tree pollen	4 (3)	11 (7.9)
Grass pollen	31 (23.3)	44 (31.7)
Weed pollen	18 (13.5)	26 (18.7)
Area of living, n (%)		
Urban	133 (100)	139 (100)
Coastal zone	133 (100)	139 (100)

a Significantly more frequent in children than in adults (*p* = 0.034; χ^2^ test).

The first cross-sectional exploratory part of the study was conducted at the Department of Medical Immunology of Beni-Messous Teaching Hospital Center in Algiers (Algeria) between June 2018 and June 2022. Clinical and epidemiological investigation of patients was performed by trained physicians, and serum samples were collected after the written informed consent was obtained. Data were compiled into a structured database by a trained investigator. Demographic, clinical and serological data generated as part of standard medical care were incorporated into a database. The dataset was independently reviewed by a second qualified investigator to ensure accuracy and completeness, and any discrepancies were resolved by cross-checking the original records. As part of standard medical care, patients with clinical suspicion of allergic disease were therefore referred for specific IgE (sIgE) testing to a panel of commercial allergen extracts guided by clinical history. Due to the lack of registered allergen extracts in Algeria, skin prick testing (SPT) could not be performed. Measurements of allergen extract-specific IgE were performed using the IMMULITE 2000 XPI® system (Siemens Medical Solutions Diagnostics, Los Angeles, CA), with a positivity threshold of ≥0.35 kUA/L to allergen extracts. One thousand and thirty-eight patients were classified as allergic based on the integration of clinical data and sIgE sensitization to the putative causal allergens (Fig. 1). Patients with incomplete clinical data, insufficient serum samples, or those who were on immunotherapy at their admission were excluded (Fig. 1). At the end, 539 patients were ultimately included for microarray-based measurement of sIgE within the framework of the present study. The flow-chart of the first exploratory study is presented in Fig. 1 and characteristics of the allergic children and adults investigated are summarized in Table 1. No sample size calculation was performed for the exploratory study.

To investigate whether the age-dependent differences regarding molecular IgE sensitization profiles could be reproduced in an independent cohort, we conducted a second molecular IgE sensitization profiling in a prospective cross-sectional study comprising 272 Turkish patients with respiratory allergic diseases, including 133 children and 139 adults (Fig. 1, Table 2). The second part of the study enrolled approximately half the number of patients included in the Algerian cohort, which, according to a previous study conducted in Türkiye appeared to be sufficient to detect differences regarding sensitizations to respiratory allergens.^30^ These participants were recruited from pediatric and adult allergy outpatient clinics in İstanbul and Samsun, Türkiye—two coastal cities with climatic conditions comparable to those of Algiers. This second “validation” cohort was recruited, with the objective to assess whether similar sensitization patterns, particularly for respiratory allergens, could be observed in another population from another continent with comparable climate and allergen exposome. Turkish patients were characterized according to the local standard medical care. Unlike patients from Algiers, Turkish patients underwent SPT instead of allergen extract-based serology using standardized commercial aeroallergen extracts (Lofarma S.p.A., Milan, Italy) in the respective departments according to the manufacturer's recommendations. The allergen panel encompassed the following indoor and outdoor aeroallergens: mold mixture (*Alternaria alternata, Chaetomium globosum, Cladosporium spp., Helminthosporium sativum*), cat and dog dander, and house dust mites (*Dermatophagoides pteronyssinus* and *D. farinae*), tree pollen mix (*Alnus incana* and other members of the *Betulaceae* family), weed pollen mix (*Artemisia vulgaris, Solidago virgaurea, Helianthus annuus, Xanthium strumarium*, and *Iva axillaris*), and grass pollen mix (*Dactylis glomerata, Festuca elatior, Lolium multiflorum, Phleum pratense*, and *Poa pratensis*). SPT positivity was defined as a mean wheal diameter of ≥3 mm compared with the negative control, 0.9% NaCl.

### Micro-array-based IgE-antibody measurements

The majority of allergen molecules, included in the experimental micro-array used for the IgE reactivity profiling of sera from the two step study described above, were recombinant allergens (n = 89) expressed either in *Escherichia coli* strain BL21-Gold (DE3) competent cells (Agilent Technologies, Inc, Calif, USA) #230132, *Spodoptera frugiperda* Sf9 insect cell line (ThermFisher Scientific/Gibco, Mass, USA, #11496015, RRID: CVCL_0549), Trichoplusia ni High Five™ (Hi5) insect cell line (ThermFisher Scientific/Gibco, Mass, USA, #B85502, RRID: CVCL_C190) or mammalian cells, HEK 293 (Expi293F™ cells; ThermFisher Scientific/Gibco, Mass, USA, #A14527, RRID: CVCL_D615). Insect cells and mammalian cells had been tested by the manufacturer for being mycoplasma-free. They were always freshly bought and expanded for the experiments. The sequences of allergen molecules were based on those deposited in the WHO/IUIS Allergen Nomenclature Database (https://allergen.org/). Glycosylation sites in allergen-encoding DNAs used for insect cell expression were mutated to prevent IgE binding to carbohydrates for allergens produced in house (Table S1). The concentration of recombinant proteins was measured by bicinchoninic acid assay (Thermo Fisher Scientific/Pierce, Mass, USA). The molecular sizes of purified allergen molecules were analyzed using SDS-PAGE and mass spectrometry (AXIMA MALDI-MS, Kyoto, Japan) and the purity of the recombinant proteins was higher than 90% by SDS-PAGE. Protein secondary structure was confirmed through Circular Dichroism spectroscopy (Jasco J-810 spectropolarimeter. Japan Spectroscopic, Tokyo, Japan). IgE reactivity and allergenic activity were assessed through IgE-binding assays and basophil activation assays, respectively. In Table S1, recombinant allergens were identified by “r” and allergens purified from natural allergen sources were identified by “n”. The table also provides details on the production of the allergen molecules and other included molecules (e.g., cross-reactive carbohydrate determinant (CCD) marker) included in the micro-array. The CCD marker HRP (horseradish peroxidase) is a commercial product purchased from Sigma–Aldrich (Missouri, USA; cat. Number: P8375). It is a purified peroxidase isolated from horseradish roots (*Armoracia rusticana*), that carries an Asn-linked oligosaccharide (N-glycan) with xylose and a fucose linked α-1,3 to the innermost GlcNAc residue (now called MMXF).

For the production of the experimental allergen microarray, glass slides containing six microarrays surrounded by an epoxy frame (Paul Marienfeld GmbH & Co. KG, Lauda-Königshofen, Germany) were coated with MCP-2 (Lucidant Polymers, Sunnyvale, CA, USA), an amine–reactive complex organic polymer which facilitates the binding of proteins and peptides. The spotting conditions, buffers and concentrations for obtaining round-shaped dots of comparable size were optimized for each protein/peptide before final spotting. For the final microarray printing, approximately 300 pL of each allergen (c = 0.5 mg/mL), dissolved in phosphate buffer (75 mM sodium phosphate, pH 8.4), were spotted as single droplet in triplicates at 500 μm horizontal and 400 μm vertical spacing using the microarray printer SciFlexArrayer (Scienion AG, Berlin, Germany). After spotting, the allergens on the slides were immobilized in a 75% humidity chamber at room temperature overnight and then incubated with blocking buffer (30 mmol/L ethanolamine in PBS containing 0.1% Tween-20). Thereafter, the allergens were stabilized with liquid plate sealer (CANDOR Bioscience GmbH, Wangen, Germany), then slides were dried by centrifugation (200 g, 1 min) in a Sigma 2–7 centrifuge using a MTP-11113 rotor (Sigma Laborzentrifugen GmbH, Osterode am Harz, Germany) and stored under vacuum at 4°C until use.

For measurement of allergen-specific IgE reactivity, slides containing allergen micro-arrays were first washed with washing buffer (PBS containing 0.1% Tween-20) and dried by centrifugation (100 g, 3 min). Subsequently, aliquots of 30 μL of undiluted serum samples were incubated with the arrays for 2 h at 22°C. For calibration and detection of background signals a calibrator serum (serum pool containing defined IgE levels as determined by ImmunoCAP measurements to several allergens) and a negative control (sample diluent, Thermo Fisher Scientific/Phadia, Uppsala, Sweden) were included in each analysis run. Then, slides were washed with washing buffer to remove unbound proteins and incubated with 30 μL of a mouse monoclonal anti-human IgE antibody (1.8 μg/mL) (Roche, Basel, Switzerland) (MABM-7H8 IgG Catalog #11988204103; https://custombiotech.roche.com/global/en/products/cb/mab-ige-m-7h8-igg-3795191.html) conjugated with DyLight™ 550-2xPEG NHS Ester (Thermo Fisher Scientific, Waltham, Massachusetts, USA) in the dark for 30 min at room temperature. The manufacturer of the monoclonal anti-IgE antibody has ensured lack of cross-reactivity with IgG, IgA and IgM. This was confirmed for the micro-array by testing for reactivity with spotted isotypes. Unbound antibodies were removed by washing with washing buffer followed by distilled water. The slides were then dried by centrifugation before scanning.

Fluorescence signals were detected with a TECAN Power Scanner (Tecan GmbH, Grödig, Austria) at 25% laser power and 50% photomultiplier (PMT) gain for IgE measurements. Image analysis was performed using MAPIX microarray image acquisition and analysis software, version 8.5.0 (Innopsys, Carbonne, France). Conversion of the measured fluorescence units to ISAC standardized units (ISUs) was based on a calibration curve generated using known allergen-specific antibody levels in the calibrator serum pool. Laboratory analyses were performed by trained staff, blinded regarding the samples. The Coefficients of Variation (CV) within one lot for measurements performed on different days were 5–7% and those from lot to lot were 9–14%, depending on IgE levels.

The results were expressed as ISAC standardized units (ISU-E, 0.3–150 ISU-E range). In this study, subjects were considered sensitized to an allergen if sIgE levels were ≥0.3 ISU-E and the results have been represented in three categories: low ([0.3–1] ISU-E), medium ([>1–15] ISU-E), and high (>15 ISU-E) sensitization.^31^

### Ethics

The study was conducted in strict adherence to the ethical principles set forth in the 2024 Declaration of Helsinki. The first exploratory cross-sectional part of the study was approved by the institutional ethics committee of Beni-Messous Teaching Hospital Center, Algiers, Algeria (15/CE/2022) whereas the second prospective cross-sectional part of the study was approved by the corresponding committees at the participating centers in Türkiye (SBA 25/814). Pseudonymized serum samples were then tested for the molecular IgE reactivity profile at the Department of Pathophysiology and Allergy Research, Medical University of Vienna, Austria, with permission of the Ethics Committee of the Medical University of Vienna (EK 1641/2014). Written informed consent was obtained from all participants or their legal guardians.

### Statistics

Statistical analysis was primarily descriptive, aiming to characterize molecular IgE sensitization profiles within the study population. Results are given in medians, absolute (n), or relative numbers (%), as appropriate. Comparative analyses were performed between pediatric and adult patients, to explore potential age-related differences in molecular IgE sensitization patterns. In order to account for a potential confounding effect of sex, comparisons of children and adults were adjusted for sex by inclusion of sex and the interaction between age and sex in the models. Logistic regression analysis has been performed to assess differences in prevalence of sensitization to the different allergens. After log-transformation of the IgE levels (i.e., for comparison of specific IgE levels) to obtain normality of residuals and homogeneity of variances, we applied an analysis of variance testing for the main effect of age. Differences in the prevalence of asthma between pediatric and adult patients were assessed using Chi-square test (χ^2^). A *p* < 0.05 was considered statistically significant. Data were analyzed by Stata 17.0 (StataCorp, College Station, TX).

### Role of funders

The funders had no role in the design of the study; in the data collection, analyses, or interpretation of data; in the writing of the manuscript; or in the decision to publish the results.

## Results

### Demographic and clinical characteristics of allergic patients

A total of 539 subjects with confirmed allergic disease were enrolled in the first exploratory cross-sectional study performed in the Algiers region, including a group of 259 children (164 males, 95 females) with a median age of 8 years (IQR: 4–11), and a group of 280 adults (123 males, 157 females) with a median age of 32 years (IQR: 26–43) (Fig. 1, Table 1). According to clinical characterization and sIgE sensitization profile, patients presented one or more of the following four allergic phenotypes: respiratory allergy, food allergy, hymenoptera venom allergy, and latex allergy. The hierarchy of the main allergy phenotypes was comparable between the two age groups, with respiratory allergies being the most prevalent, followed by food and hymenoptera venom allergies. Interestingly, asthma but not rhinitis was significantly more common in children than in adult patients with respiratory allergy (*p* < 0.001; χ^2^ test) (Table 1). A detailed description of the study population is presented in Table 1. The distribution of participants according to area of living was comparable between children and adults. In both groups, the majority lived in urban rather than rural regions (88.2% *vs* 11.8% for children, and 89.8% *vs* 10.2% for adults). Additionally, most participants were primarily living in coastal areas (98% for children and 96% for adults).

In the second prospectively collected cross-sectional Turkish cohort, 272 patients were included, comprising 133 children (83 males, 50 females) and 139 adults (60 males, 79 females) (Fig. 1). The median age was 7.7 years (IQR: 6.2–9.8) in children and 32 years (IQR: 25–41) in adults. All patients had respiratory allergies such as allergic rhinitis or asthma and came from an urban environment (Table 2). Asthma was found to be significantly more common among children than adults (75.2% *vs* 63.3%; *p* = 0.034; χ^2^ test). Demographic and clinical characteristics of the Turkish cohort are given in Table 2.

### Molecular IgE sensitization profiles are similar in the Algiers and Turkish cohort and dominated by IgE sensitization to allergens from *D. pteronyssinus*

The highest sensitization prevalence in the whole Algiers cohort of patients was found to allergens from *D. pteronyssinus* (Fig. 2) (Der p 23, Der p 1, Der p 2, Der p 5 and Der p 21) with the highest median IgE levels (Fig. 2). The following allergen molecules were next in the hierarchy in the whole population: major olive pollen allergen Ole e 1 (21.5%), *Blomia tropicalis* allergens Blo t 21 and Blo t 5 (19.5% and 18.4%) and major cat allergen Fel d 1 (18.4%) (Fig. 2). Notably, 15% of the patients were reactive to CCDs. In the group with respiratory allergy (n = 417), sensitization was dominated by the three major allergens from *D. pteronyssinus*: Der p 23 (65%), Der p 1 (58.3%) and Der p 2 (57.1%), which also showed the highest IgE levels (Fig. 3A, Table S2).

**Fig. 2:**
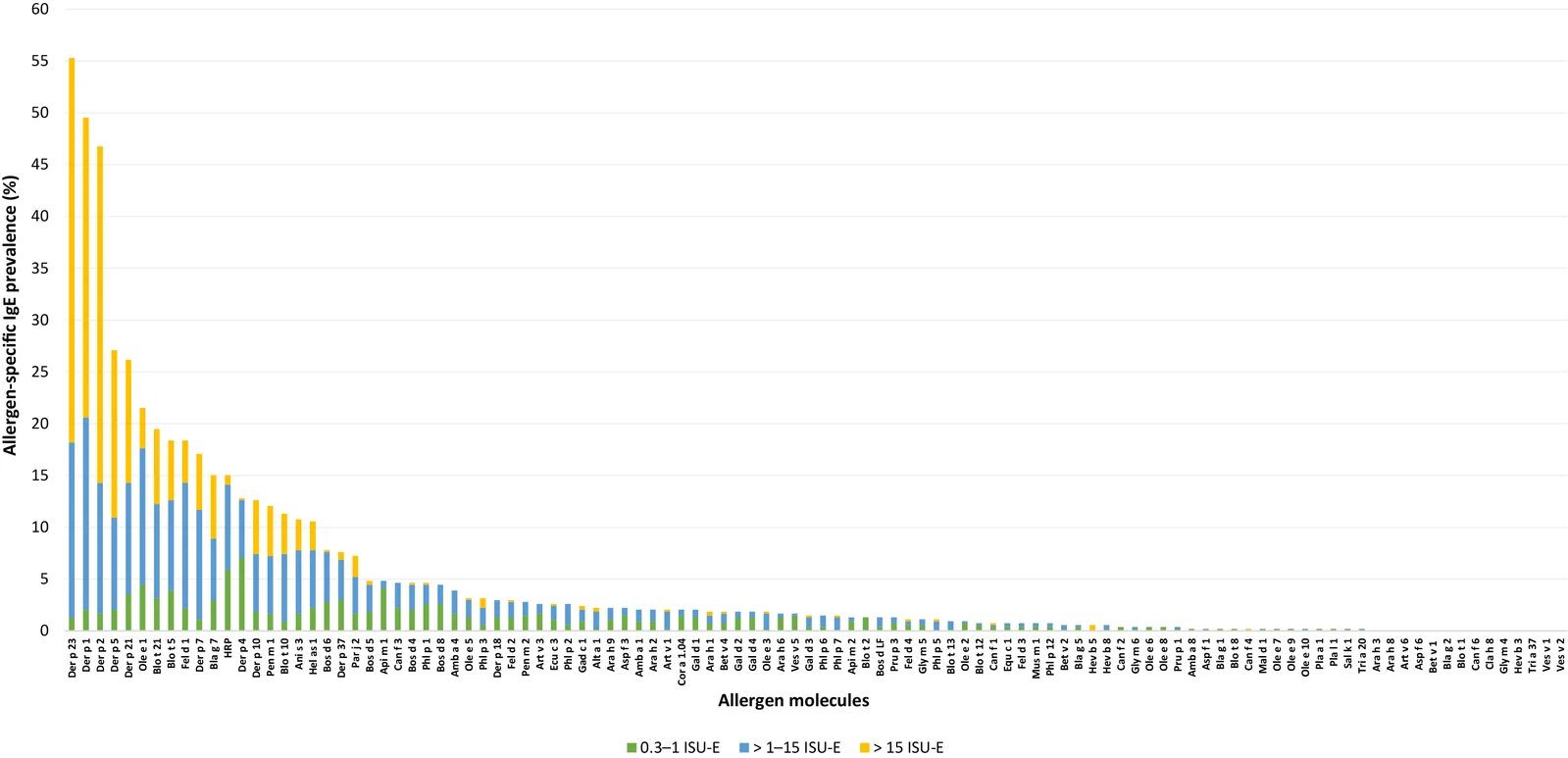
Hierarchy of sensitization to molecular allergens in the full Algiers cohort (N = 539) according to frequencies of IgE recognition. Shown are percentage frequencies of IgE recognition (y-axis) and allergen-specific IgE levels (0.3–1 ISU-E: green; >1–15 ISU-E: blue and >15 ISU-E: yellow) for each positive allergen (x-axis).

**Fig. 3:**
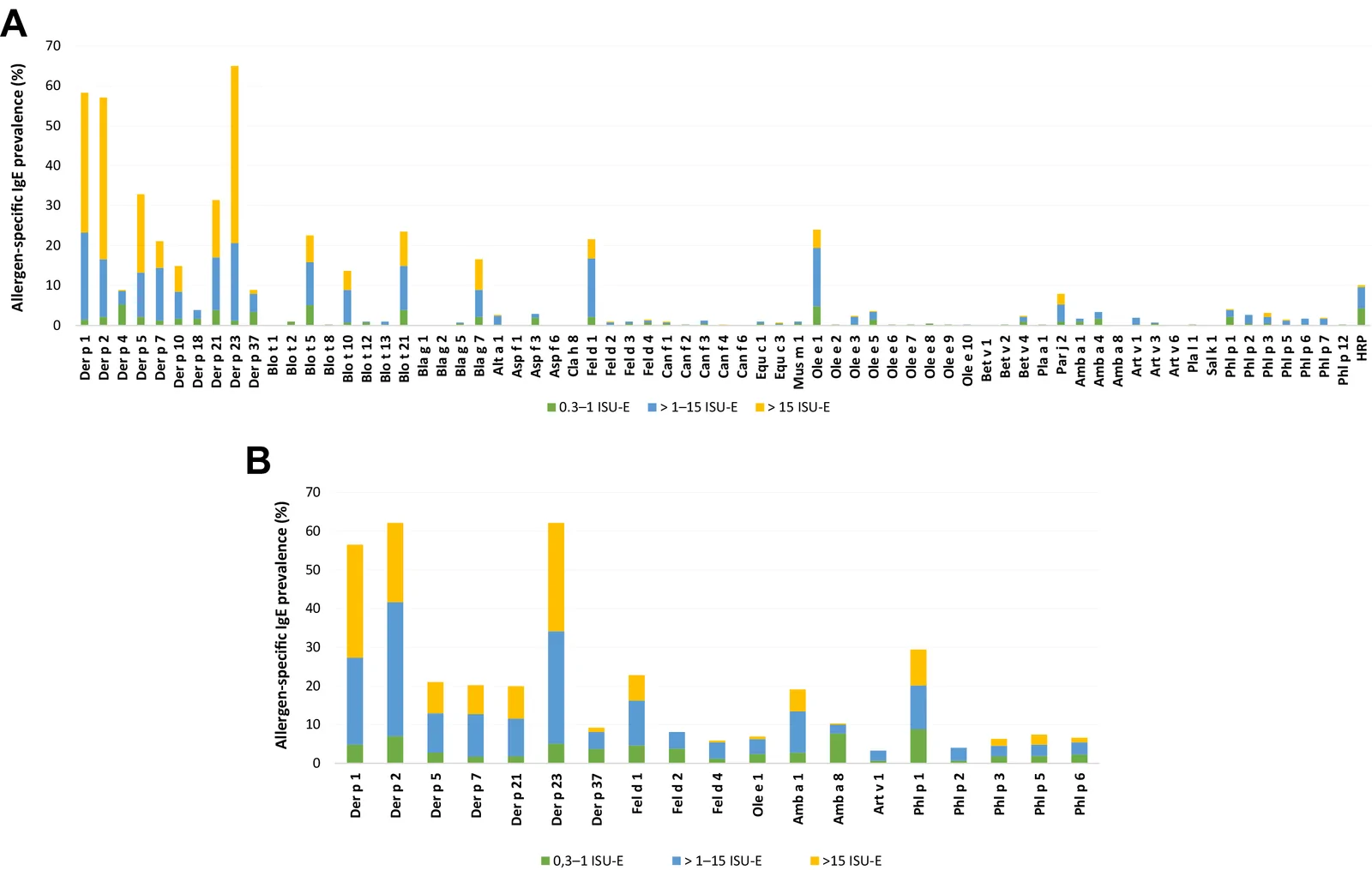
Molecular sensitization to aeroallergens in the respiratory-allergy cohorts from (**A**) Algiers (N = 417) and (**B**) Türkiye (N = 272). Shown are percentage frequencies of IgE recognition (y-axes) and allergen-specific IgE levels (0.3–1 ISU-E: green; >1–15 ISU-E: blue and >15 ISU-E: yellow) for each positive allergen (x-axes). HRP, horseradish peroxidase.

In total, 198 subjects of the whole Algiers patients (n = 539) had IgE against at least one of the following cross-reacting molecules: tropomyosins (84/539), nsLTP (58/539), serum albumins (44/539), seed storage proteins (26/539), lipocalins (14/539), PR10 proteins (13/539), 2-EF hands calcium-binding proteins (11/539) and profilins (5/539) (Table S3). Co-sensitization to more than one molecule clearly prevailed within tropomyosins, 2 EF-hands calcium-binding proteins and profilins while mono-reactivity was more frequent among nsLTP (mainly Par j 2), PR10 allergens (mainly Cor a 1.04) and lipocalins (mainly Fel d 4) (Table S3).

Molecular analysis of the Turkish cohort (Table S4) revealed a sensitization profile to respiratory allergens similar to that of the Algerian cohort (Table S2), characterized by a predominance of house dust mite (HDM) allergens in patients with respiratory allergy. The highest sensitization rates and median IgE levels were recorded for Der p 23 (62.1%), Der p 2 (62.1%), and Der p 1 (56.6%) (Fig. 3B). These were followed by sensitizations for Phl p 1 (29.4%) and Fel d 1 (22.8%).

### Dominant outdoor allergens in patients with respiratory allergy in the Algiers and Turkish cohort

Among outdoor respiratory allergens, olive pollen was the most important allergen source with Ole e 1 being recognized by 24% of the subjects in Algiers cohort (Fig. 3A, Table S2). No patient reacted to the major birch allergen Bet v 1 and the reactivity to the minor allergens Bet v 2 and Bet v 4 was mainly related to cross-reactivity among profilins and 2 EF-hands calcium binding-proteins (Table S3). *Parietaria* was the most important weed pollen allergen source as indicated by the major allergen Par j 2 (7.9%), whereas the other weed pollen allergens were less frequently recognized (Fig. 3A, Table S2). Grass pollen reactivity was dominated by the major timothy grass pollen allergen Phl p 1, but overall sensitization rates remained below 5%. Interestingly, IgE sensitizations against the carbohydrate marker HRP was quite common among patients with respiratory allergies (10.1%). In the Turkish cohort, Phl p 1 (29.4%) was the most prevalent outdoor allergen molecule, followed by Amb a 1(19.1%), Amb a 8 (10.3%) and Ole e 1 was recognized by 6.9% of the patients (Table S4, Fig. 3B).

### Children are predominantly sensitized to HDM allergens with higher specific IgE levels, whereas adults show more frequent sensitization to outdoor allergens

In the exploratory Algiers cohort of patients with respiratory allergy we investigated whether age may have an impact on the sensitization pattern to molecular aeroallergens. As illustrated in Fig. 4A, Table S2 and Figure S1, children had significantly higher frequencies of sensitization to important *Dermatophagoides* allergens (i.e., Der p 1: *p* < 0.001; Der p 2: *p* < 0.001; Der p 5: *p* < 0.001; Der p 7: *p* = 0.002; Der p 18: *p* < 0.001; Der p 21: *p* = 0.001; Der p 23: *p* = 0.001; logistic regression adjusted for sex and the sex × age interaction) compared to adults. Also, higher IgE levels to HDM allergens were observed in children than in adults (Figure S1). No significant difference was found in the IgE reactivity to *Blomia* allergens between adults and children (Fig. 4A, Table S2). Sensitization to the cat allergen Fel d 1 was more frequent and associated with higher IgE levels in children compared to adults but the difference did not reach statistical significance (Fig. 4A, Table S2).

**Fig. 4:**
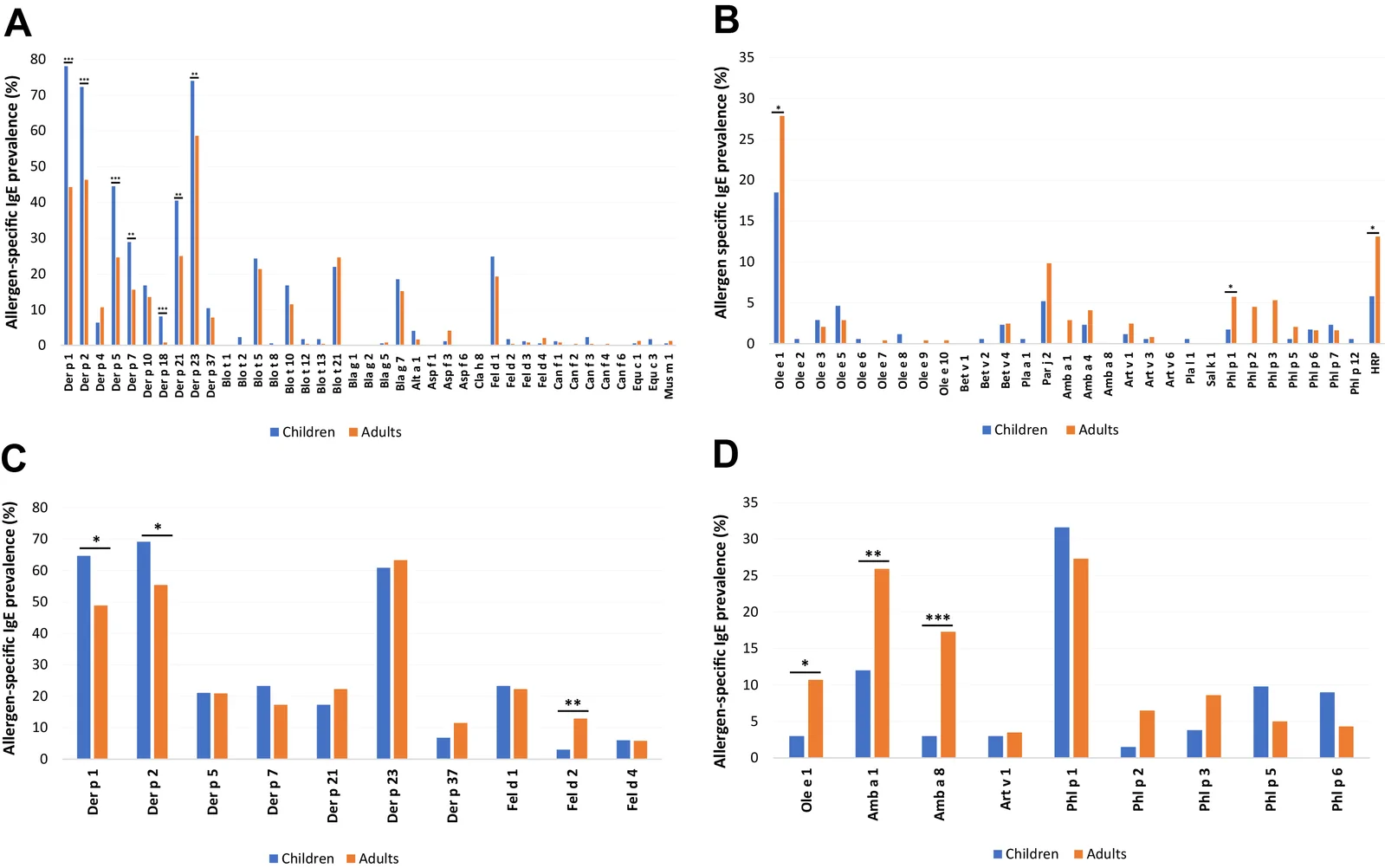
Age-stratified molecular sensitization to (**A, C**) indoor and (**B, D**) outdoor aeroallergens in children (N = 173; blue bars) and adults (N = 244; orange bars) with respiratory allergy from Algiers (**A, B**) and children (N = 133; blue bars) and adults (N = 139; orange bars) with respiratory allergy from Türkiye (**C, D**). Shown are percentage frequencies (y-axes) of IgE recognition for each positive respiratory allergen (x-axes). *p* value ∗ <0.05 ∗∗ <0.01 ∗∗∗ <0.001 (*p* values were obtained using logistic regression adjusted for sex and interaction between sex and age). HRP, horseradish peroxidase.

Conversely, the analysis of sensitization to outdoor allergens revealed an opposite pattern. IgE sensitization rates to the major pollen allergens Ole e 1 (*p* = 0.037; logistic regression adjusted for sex and the sex × age interaction) and Phl p 1 (*p* = 0.046; logistic regression adjusted for sex and the sex × age interaction) were significantly higher in adults compared to children, while a similar, non-significant trend was observed for Par j 2 (Fig. 4B, Table S2). There was no significant difference regarding IgE levels to these allergens between the two age groups (Figure S2, Table S2). Sensitization to the CCD marker HRP was also more frequent in adults (13.1% in adults *vs* 5.8% in children, *p* = 0.014, logistic regression adjusted for sex and the sex × age interaction), in line with higher sensitization rates to glycosylated outdoor allergens in this group (Table S2).

Notably, similar findings were obtained in the Turkish cohort. Sensitization to Der p 1 and Der p 2 was significantly more frequent in children than in adults (64.7% *vs* 48.9%, *p* = 0.018 and 69.2% *vs* 55.4%, *p* = 0.019; logistic regression adjusted for sex and the sex × age interaction, respectively), and median IgE levels for most HDM molecules were higher in children than in adults (Fig. 4C and Table S4). Among outdoor allergen molecules, Amb a 1 (*p* = 0.002; logistic regression adjusted for sex and the sex × age interaction), Amb a 8 (*p* < 0.001; logistic regression adjusted for sex and the sex × age interaction) and Ole e 1 (*p* = 0.016; logistic regression adjusted for sex and the sex × age interaction) showed significantly higher sensitization rates in adults compared with children (Fig. 4D, Table S4). Median IgE levels did not differ significantly between age groups for these outdoor allergens in the Turkish cohort (Table S4).

### IgE sensitization to food, venom and other allergens in the exploratory Algiers cohort

Within the exploratory Algiers cohort, 137 patients were diagnosed with food allergy (25.4%), of whom over 70% were under the age of 18. Their sensitization was predominantly directed against class 1 food allergens (Fig. 5A). The hierarchy of food molecules recognized by children differed substantially from that of adults (Fig. 5B, Table S5). The cow's milk allergens (Bos d 4, Bos d 5, Bos d 8 and Bos Lf) and egg allergens (Gal d 1, Gal d 2 and Gal d 3) were exclusively recognized by children. Only one adult reacted to the cow's milk allergen Bos d 6 and another one to the egg allergen Gal d 4 (Fig. 5B, Table S5). In addition, the seed storage proteins (Ara h 1, Ara h 2, Ara h 6 and Gly m 6) and the PR10 molecule Cor a 1.04 were also exclusively recognized by children. IgE sensitizations to Ara h 9, Gly m 5 and Gad c 1 were more frequent among children, whereas significantly more adults showed IgE reactivity to the shrimp allergens Pen m 1 and Pen m 2 (Fig. 5B, Table S5). Pen m 1 was predominantly recognized in adults and represented the most frequently recognized food allergen molecule among patients with food allergy (40/137), with the highest specific IgE levels. Notably, all Pen m 1 sensitized individuals were co-sensitized to Der p 10 with comparable sIgE levels (9.8 [2.7–41.1] *vs* 12.9 [4.2–48.2] ISU-E). Among these patients, 36 (90%) had clinically confirmed shrimp allergy whereas 4 (10%) had no history of shrimp consumption.

**Fig. 5:**
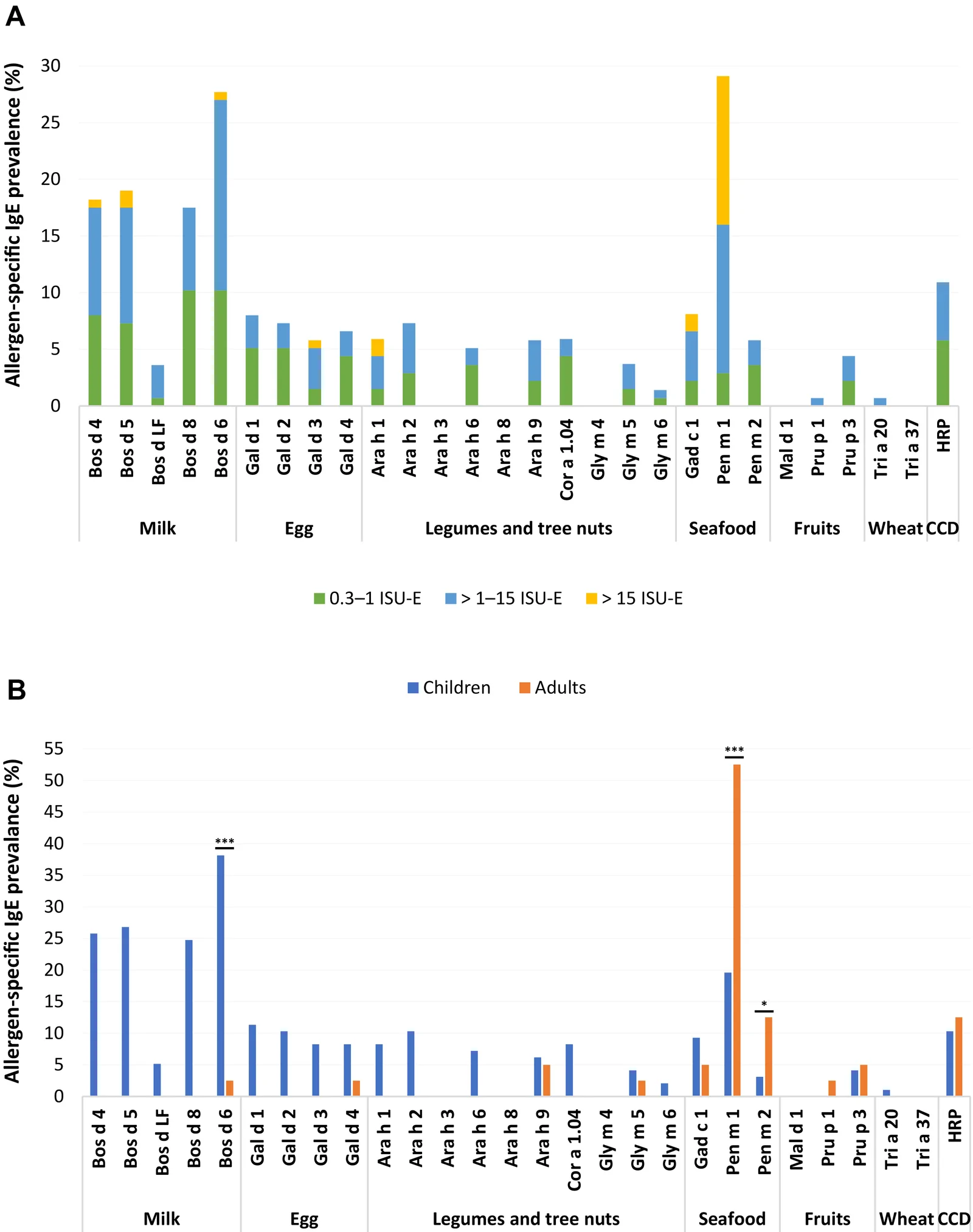
IgE sensitization profile to food allergens among patients with food allergy from Algiers (N = 137). (**A**) Percentage frequencies of IgE recognition (y-axis) and allergen-specific IgE levels (0.3–1 ISU-E: green; >1–15 ISU-E: blue and >15 ISU-E: yellow) for each positive allergen (x-axis). (**B**) Sensitization prevalences (y-axis) of children (N = 97; blue bars) and adults (N = 40; orange bars). *p* value ∗ <0.05 ∗∗ <0.01 ∗∗∗ <0.001 (*p* values were obtained using logistic regression adjusted for sex and interaction between sex and age). CCD, cross-reactive carbohydrate determinant; HRP, horseradish peroxidase.

None of the Algiers patients showed IgE reactivity to the PR10 molecules Ara h 8, Gly m 4, Mal d 1, nor to the peanut molecule Ara h 3 or to the wheat allergen Tri a 37 (Fig. 5A). Similar to patients with respiratory allergies, IgE sensitizations to CCDs were common (10.9%) among those with food allergies. Nevertheless, no significant difference between children and adults was observed.

A total of 56 patients (10.4%) in the exploratory Algiers cohort were diagnosed with hymenoptera venom allergy. As shown in Fig. 6A and Table S6, the major bee venom allergen Api m 1 was more frequently recognized than major wasp venom allergen Ves v 5 (44.6% *vs* 16.1% respectively). Sensitization to bee venom allergens was more frequent among children than in adults, while the opposite trend was observed for the wasp allergens Ves v 5 (Fig. 6B). However, the differences between the two groups were not statistically significant (Table S6). Of note, IgE reactivity to the carbohydrate marker HRP was found in more than half of the patients with hymenoptera venom allergy (62.5%) with comparable prevalence and IgE levels between children and adults (Table S6).

**Fig. 6:**
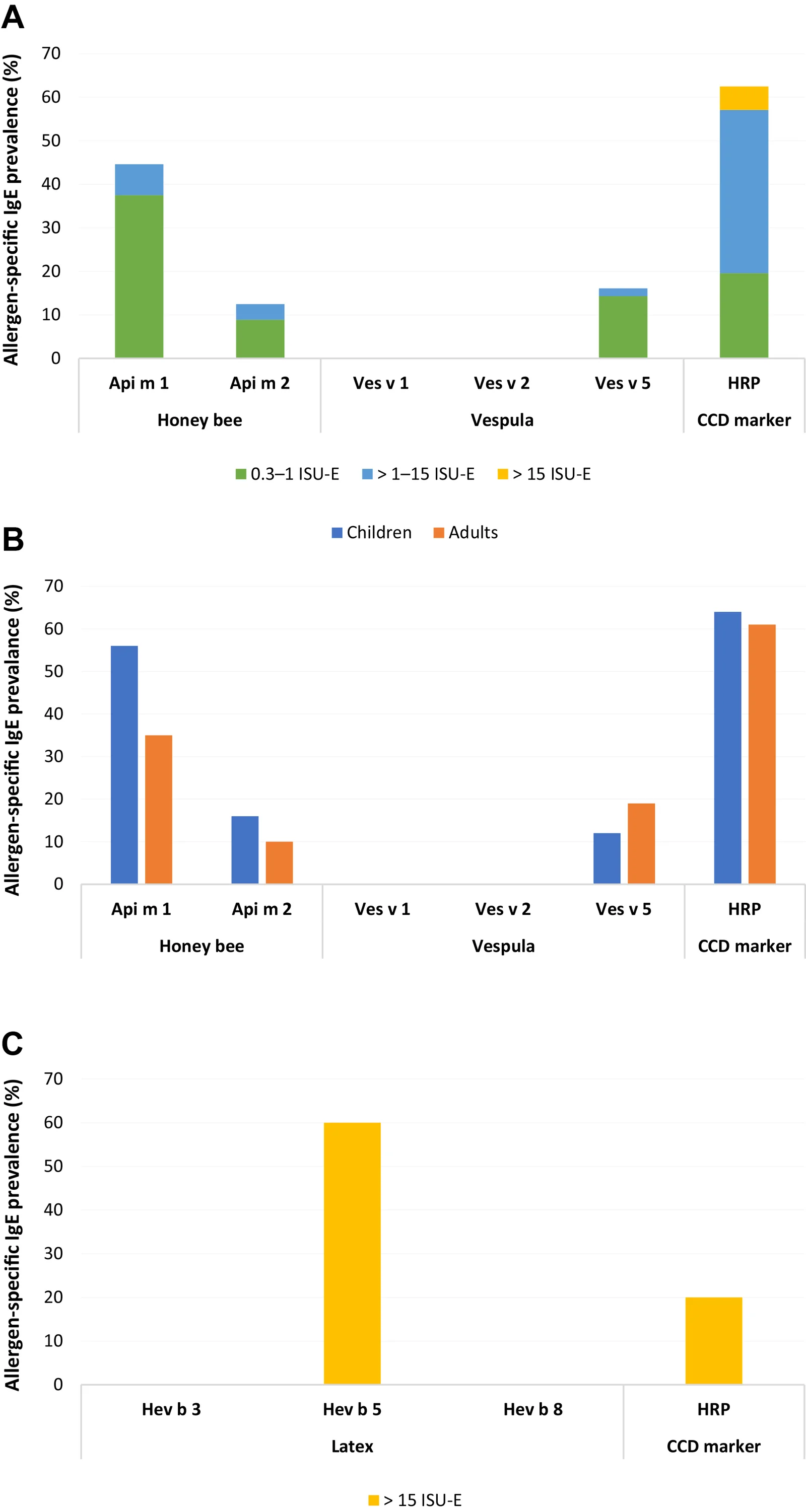
Prevalence of IgE sensitization to venom allergens among venom-allergic patients (**A, B**) (N = 56) and to latex allergens among latex-allergic patients (**C**) (N = 5) from Algiers. (**A**) percentage frequencies of IgE recognition (y-axis) and allergen-specific IgE levels (0.3–1 ISU-E: green; >1–15 ISU-E: blue and >15 ISU-E: yellow) for each positive allergen (x-axis). (**B**) Sensitization prevalences (y-axis) of children (N = 25; blue bars) and adults (N = 31; orange bars) to the allergens (x-axis). (**C**) Sensitization prevalences (y-axis) to the latex allergen molecules Hev b 3, Hev b 5, and Hev b 8 (x-axis) among five adults with latex allergy. Yellow bars indicate allergen-specific IgE levels >15 ISU-E. CCD, cross-reactive carbohydrate determinant; HRP, horseradish peroxidase.

Latex allergy was reported in only five patients in the Algiers cohort, all of whom were adults and healthcare professionals. Among the three tested latex allergen components (Hev b 3, Hev b 5 and Hev b 8), only Hev b 5 was recognized (Fig. 6C), with a sensitization rate of 60% and a median specific IgE level of 30.8 [20.4–34.6] ISU-E.

## Discussion

In the present study we investigated if molecular IgE reactivity profiles differ in allergic children and adults from two regions in different continents with similar climate and exposome. We found that significantly more children than adults with respiratory allergy were sensitized to major respiratory house dust mite allergens, whereas significantly more adults than children were sensitized to regionally common respiratory outdoor allergens. This finding may be clinically relevant because asthma occurred more frequently in children than in adult patients. The first part of our study was an exploratory molecular profiling study performed with the allergen micro-array technology developed by us using sera from allergic adults and children in Algiers. The molecular IgE profiles were newly determined and then interpreted in relation to the available clinical and demographic information. In this first part, we analyzed sera from well-characterized allergic patients across two age groups: 259 children with a median age of 8 years and 280 adults with a median age of 32 years, which allowed us to investigate possible age-related differences in molecular IgE sensitization profiles. Several important results were obtained. First, we were able to identify the most important allergen molecules in the Algiers cohort. We found a predominant sensitization to *Dermatophagoides* allergens among patients with respiratory allergy. The subjects enrolled in our study were mainly from north–central Algeria (coastal areas), where the climate is very humid, which could explain why sensitization to HDM allergens predominated in this region.^21^ Der p 23 was the dominating respiratory allergen followed by Der p 1 and Der p 2 but also other relevant HDM allergens such as Der p 5, Der p 21 and Der p 7 were frequently recognized. Our results are thus in agreement with earlier studies having identified the aforementioned allergens as the most important HDM allergens.^11^^,^^32^ Interestingly, dominance of Der p 23 sensitization was found also for South Africa, while the European and American populations more often reacted to the major group 1 and 2 allergens.^32^ However, in contrast to South Africa, where Der p 7 acts as a major allergen with 56% of sensitized patients,^32^ the latter remains a mid-tier allergen in our population. Thus, it depends also on the geography whether an allergen can be considered as “major” or “minor”. This finding has important implications for AIT since it has been shown that commercially available allergen extract-based HDM allergy vaccines induce IgG responses mostly to Der p 1 and Der p 2, less often to Der p 23, and to a lesser extent to other allergens.^33^^,^^34^ IgE sensitization to Blo t 21 and Blo t 5 was also common in the Algerian population, but it is quite possible that this is due to partial cross-reactivity with the corresponding HDM allergens Der p 21 and Der p 5 which were recognized by more patients and IgE levels against the HDM allergens tended to be higher. In fact, it has recently been shown that *B. tropicalis* and HDM are antigenically different allergen sources as far as group 2, 5 and 21 allergens are concerned.^35^ At present, group 1, 7 and 23 allergens from *B. tropicalis* remain incompletely characterized. Nevertheless, available data indicate that some of these allergens exhibit IgE reactivity^36^ and limited cross-reactivity with HDM allergens.^37^

The major cat allergen, Fel d 1 was the other dominating indoor respiratory allergen whereas sensitization to allergens from other pets, including dogs, was not common and mostly occurred alongside cat allergen reactivity. These data strongly mirrored the Algerian community's habits where cat ownership is common while dogs are less often kept indoor due to religious beliefs. Indeed, it has been shown that reactivity to dog allergens was closely related to owning a dog, whereas sensitization to Fel d 1 was common and often occurs even without possessing a cat, presumably due to its high allergenicity and its ubiquitous presence in the human environment.^38^ Sensitization to dog allergens seems to be more prevalent in industrialized countries where on average 30% of all households have a dog.^39^ Based on the sensitization frequencies observed among the four tested cat molecular allergens, Fel d 1 emerges as the primary candidate for inclusion in immunotherapy formulations targeting cat allergic patients in the Algiers population. In contrast, for other populations, allergens such as Fel d 4 and Fel d 7, given their higher prevalence and allergenic activity, should also be taken into consideration for specific immunotherapy of cat allergy.^40^^,^^41^

Regarding outdoor respiratory allergens, Ole e 1, Par j 2 and Phl p 1 dominated whereas other outdoor respiratory allergen molecules were of low importance, which fits the vegetation profiles in the area of the study.^42^ To some extent, the molecular IgE sensitization profile to pollen allergens in Algiers was similar to that reported earlier for southern parts of Italy,^43^ which may be explained by a similar plant exposome.

According to the molecular IgE sensitization profiles we could clearly identify a handful of important respiratory allergen sources comprising HDM, cat as well as pollen from olive, *Parietaria* and grass which are needed to cover AIT-approaches in the Algiers region, and for the future development of allergen-specific preventive approaches. Our study therefore exemplifies the importance of molecular IgE reactivity profiling for the establishment of systematic allergen-specific treatment approaches fitting the needs of certain regions and/or countries.

In addition to respiratory allergens, we also investigated IgE reactivity to food allergens. We found that IgE sensitization to class 1 food allergens was dominated by allergens from milk, egg, peanut, as well as by sensitization to the major fish allergen, Gad c 1 and the major shrimp allergen, Pen m 1. Children mainly reacted to cow's milk and egg white allergens, which are the two earliest foods given to babies and both are commonly consumed by children. These findings are consistent with the data of Jerbi et al.^22^ on the sensitization of the children in Tunisia, a neighboring country. Peanut seed storage proteins were exclusively recognized by children, Ara h 2 was the most prevalent followed by Ara h 1 and Ara h 6. A comparable rate of positivity has been found for the nsLTP Ara h 9, underlining the importance of including Ara h 9 in the diagnosis of peanut allergy like for the other Mediterranean countries (i.e., South Europe).^43^ This point seems particularly relevant for the Tunisian population, where all the children sensitized to peanut extract reacted to the nsLTP Ara h 9, and only one case was co-sensitized to the storage protein Ara h 3.^22^ For adults, the food allergy profile was dominated by IgE reactivity to the shrimp allergens Pen m 1 and Pen m 2. The tropomyosin from shrimp, Pen m 1 is known to show high IgE cross-reactivity with allergens in HDM and cockroach.^44^^,^^45^ Among the overall population, 65 subjects were positive for Pen m 1 (Fig. 2), all of them were also positive for Der p 10 and allergic to HDM. More than half of these patients (36/65) had clinically confirmed shrimp allergy, while the others had no history of shrimp consumption. These findings highlight the relatively frequent occurrence of shrimp allergy in our population, which may be explained by the fact that most of the investigated patients live close to the sea.

A central finding of our study was that the molecular IgE sensitization profiles to indoor and outdoor respiratory allergens in children and adults with respiratory allergy were very different. Significantly more children were sensitized to major HDM allergens, Der p 1, Der p 2 and Der p 23 (*p* < 0.01; logistic regression adjusted for sex and the sex × age interaction). Asthma was also significantly more frequent in children than in adults, whereas adults showed a significantly higher prevalence of sensitization to outdoor pollen allergens common in the region, namely Ole e 1 and Phl p 1 (*p* < 0.05; logistic regression adjusted for sex and the sex × age interaction). Allergen-specific IgE sensitization profiles are established in the first few years of life^19^^,^^46^ but do not change in adults^17^ whereas allergen-specific IgE levels vary depending on repeated and cumulative allergen exposure.^47^ Nevertheless, allergen-specific IgE levels followed the trend observed for IgE sensitization profiles, being higher for indoor respiratory allergens in children than in adults and they were higher for pollen allergens in adults as compared to children. The difference regarding IgE recognition profiles in the two age groups seems to have clinical relevance because asthma was more prevalent in children (*p* < 0.001; χ^2^ test) than in adults although the latter were likely exposed to higher cumulative allergen doses than the children.

In order to study if the finding made for the Algiers region (i.e., that children and adults have different molecular IgE sensitization profiles to indoor and outdoor allergens with possible clinical implications) may be found in another region with similar climate and allergen exposome, we conducted a second prospective cross-sectional study. In this second cross-sectional study, we investigated the molecular IgE sensitization profiles in a cohort comprising 272 Turkish patients comprising 133 children and 139 adults from Istanbul and Samsun. These regions from Türkiye were selected to include another urban population from another continent with a similar climate and allergen exposome. Like in the population from the Algiers region, IgE sensitization to the major house dust mite allergens in children was significantly more frequent (Der p 1: *p* = 0.018; Der p 2: *p* = 0.019; logistic regression adjusted for sex and the sex × age interaction) whereas significantly more adults were sensitized to the prevalent outdoor allergens (e.g., Ole e 1: *p* = 0.016; Amb a 1: *p* = 0.002; Amb a 8: *p* < 0.001; logistic regression adjusted for sex and the sex × age interaction).

Importantly also, Turkish children suffered more frequently from asthma as compared to adults, indicating that the results obtained for Algeria may have some relevance at least for the investigated regions.

Prior research has established that IgE sensitization is affected by multiple endogenous and exogenous factors.^48^ Among exogenous factors, early in life contact with allergens from the environment (i.e., allergen exposome) determines the allergen sensitization profile of an allergic person.^19^ In addition, environmental factors such as hygienic conditions during birth (e.g., cesarean section^49^) and early life (e.g., rural *vs* urban environment, anthroposophic life-style) were reported to influence the general frequency of IgE sensitization.^50^^,^^51^ These insights are important when interpreting our findings, as they highlight that both the nature and timing of environmental exposures can have lasting effects on allergic sensitization patterns. In this light, one plausible explanation for the different IgE sensitization profiles in different age groups in our study would be a bias introduced by the dominance of living conditions brought about by different frequency of residence in rural *vs* urban regions. However, this possibility is highly unlikely because there was no significant difference regarding the areas of living for children and adults in the investigated populations (Table 1 and Table 2). We therefore propose several not mutually exclusive hypotheses for our results, which, however, remain speculative at this point. One possibility for the observed differences of molecular IgE sensitizations in the two age groups could be differences in lifestyle. In the last decades, indicated also by the term “generation Z”, children are usually brought up more in indoor environments and spend more time there than in the outdoor environment as compared to previous generations which spent more time in the outdoor environment.^52^, ^53^, ^54^ Climate change, particularly rising temperatures and increasingly frequent extreme weather events, may lead people to spend more time indoors.^55^, ^56^, ^57^, ^58^ In addition, changes in vegetation patterns resulting from climate change and urban landscaping, together with historical differences in environmental exposure, may have contributed to the observed sensitization patterns.^18^ Furthermore, other parameters such as different levels of pollution,^59^ diet change, global warming per se and humidity changes which may have occurred between now and decades ago may have affected our findings.

It is important to mention the lack of skin testing in the exploratory cohort as a major limitation of our study, due to the unavailability of registered allergen extracts in Algeria. It is another limitation of our study that we did not have longitudinal data to permit reconstruction of the history of allergy development in the investigated patients, although molecular IgE sensitization profiles do not change in a relevant manner in adulthood.^17^ Yet another limitation of our study is that it is a descriptive cross-sectional study. An unambiguous causal relationship between the observed IgE sensitization profiles and the occurrence of certain symptoms such as asthma can therefore not be established. However, it is a strength of our study that we could perform a meticulous molecular IgE reactivity profiling with a high-resolution micro-array technology in a relatively large cohort of patients. Furthermore, we were able to achieve data supporting the results obtained in the first exploratory part of the study in a second part of the study comprising a prospective cohort from another continent with similar climate and exposome.

The results of our study have direct implications for allergen-specific forms of treatment and prevention and clearly highlight the value of systematic molecular IgE reactivity profiling in different regions of the world. In addition, our findings may warrant future studies investigating changes of molecular allergen recognition profiles and possible related changes of clinical phenotypes in complex longitudinal cohort studies which, for example, may involve nested birth cohorts, which are started approximately every 10–20 years with accompanying monitoring of exogenous parameters capable of affecting specific molecular sensitization profiles. Even though our studies were limited to only two regions in Algiers and Türkiye, the findings suggest that environmental and lifestyle-related factors may indeed contribute to shaping allergen sensitization profiles and associated clinical manifestations in populations.

## Contributors

LLM, SV, and RV designed the study. OA, MRB, KWC, RC, MDC, MC, YD, ND, AD, MFT, PG, HJH, EK, AKr, ES, DT, and MW expressed, purified and characterized allergens. HG, AKar, BL, and CP supervised allergen production. MT and HJH produced allergen chip and/or performed quality control of allergens on chips. LLM performed the chip analysis for the Algerian cohort. LLM and SV accessed and verified the underlying data reported in the manuscript. LLM, SV, RV, and RD analyzed and interpreted the data. LLM, BB, and RD conducted the initial laboratory investigations for patient diagnosis. LS, NB, and MG recruited patients and provided clinical data in Algeria. LLM, SV, and RV wrote the first draft of the manuscript. AKaz, RS, MHC, and BES recruited Turkish patients and provided clinical data in Türkiye. LL performed the chip analysis for the Turkish cohort. All authors critically reviewed the manuscript and approved the submitted version.

## Data sharing statement

The raw data supporting the conclusions of this article are available from the corresponding author upon reasonable request. Serological, sex, age and clinical data and the analysis code will be made available after signing of a Material Transfer Agreement as pseudonymized data in a protected manner.

## Declaration of interests

Rudolf Valenta serves as a lecturer for HVD Life Science. The rest of the authors report no conflict of interest. The authors with a Russian affiliation declare that they have prepared the article in their “personal capacity” and/or that they are employed at an academic/research institution where research or education is the primary function of the entity.
