# Supplementary material for: Age-related molecular IgE sensitization to indoor and outdoor respiratory allergens: a two-step cross-sectional study in Algeria and Türkiye
Source: eBioMedicine. 2026 Sep 24;132:106484. doi: 10.1016/j.ebiom.2026.106484 (PMC13688041; doi:10.1016/j.ebiom.2026.106484)
Supplement: Supplementary file 1 [file mmc1.docx]

**Table S1.** List of allergens contained on the allergen-chip.

| **No.** | **Allergen-source** | **Allergen** | **R/N** | **Route of exposure** | **Biochemical name/Allergen family** | **Source** |
| --- | --- | --- | --- | --- | --- | --- |
| 1 | *Birch* | Bet v 1 | R | inh | PR-10 | Own-production |
| 2 |  | Bet v 2 | R | inh | Profilin | Biomay, Vienna, Austria |
| 3 |  | Bet v 4 | R | inh | 2 EF-hands calcium binding protein | Own-production |
| 4 | *Olive* | Ole e 1 | R | inh | Common olive group 1 | Own-production |
| 5 |  | Ole e 2 | R | inh | Profilin | Own-production |
| 6 |  | Ole e 3 | R | inh | 2 EF-hands calcium binding protein | Own-production |
| 7 |  | Ole e 5 | R | inh | Superoxide dismutase [Cu-Zn] | Own-production |
| 8 |  | Ole e 6 | R | inh | Oleaceae group 6 | Own-production |
| 9 |  | Ole e 7 | R | inh | nsLTP, type 1 | Own-production |
| 10 |  | Ole e 8 | R | inh | 4 EF-hands calcium binding protein | Own-production |
| 11 |  | Ole e 9 | R | inh | 1 3-beta glucanase | Own-production |
| 12 |  | Ole e 10 | R | inh | X8 domain containing protein | Own-production |
| 13 | *Plane tree* | Pla a 1 | R | inh | Invertase Inhibitor | Own-production |
| 14 | *Timothy grass* | Phl p 1 | R | inh | Grass group 1 (Beta-Expansin) | Biomay, Vienna, Austria |
| 15 |  | Phl p 2 | R | inh | Grass group 2/3 | Own-production |
| 16 |  | Phl p 3 | R | inh | Grass group 3 | Own-production |
| 17 |  | Phl p 5b | R | inh | Grass group 5 | Own-production |
| 18 |  | Phl p 6 | R | inh | Grass group 5/6 | Own-production |
| 19 |  | Phl p 7 | R | inh | 2 EF-hands calcium binding protein | Own-production |
| 20 |  | Phl p 12 | R | inh | Profilin | Biomay, Vienna, Austria |
| 21 | *Ragweed* | Amb a 1 | R | inh | Pectate lyase | Own-production |
| 22 |  | Amb a 4 | R | inh | Defensin-like protein | Own-production |
| 23 |  | Amb a 8 | R | inh | Profilin | Own-production |
| 24 | *Mugwort* | Art v 1 | R | inh | Defensin-like protein | Own-production |
| 25 |  | Art v 3 | R | inh | nsLTP, type 1 | Own-production |
| 26 |  | Art v 6 | R | inh | Pectate lyase | Own-production |
| 27 | *Plantain* | Pla l 1 | R | inh | Ole e 1-related protein | Own-production |
| 28 | *Wall pellitory* | Par j 2 | R | inh | nsLTP, type 1 | Own-production |
| 29 | *Saltwort* | Sal k 1 | R | inh | Pectin methylesterase | Own-production |
| 30 | *Latex* | Hev b 3 | R | inh, skin | Small rubber particle protein | Biomay, Vienna, Austria |
| 31 |  | Hev b 5 | R | inh, skin | Acidic protein | Own-production |
| 32 |  | Hev b 8 | R | inh, skin | Profilin | Own-production |
| 33 | *Alternaria* alternata | Alt a 1 | R | inh | Acidic glycoprotein | Own-production |
| 34 | *Aspergillus fumigatus* | Asp f 1 | R | inh | Mitogillin family | Own-production |
| 35 |  | Asp f 3 | R | inh | Peroxysomal protein | Own-production |
| 36 |  | Asp f 6 | R | inh | Mn superoxide dismutase | Own-production |
| 37 | *Cladosporium*  *herbarum* | Cla h 8 | R | inh | Mannitol dehydrogenase | Biomay, Vienna, Austria |

**Table S1.** (Continued)

| **No.** | **Allergen-source** | **Allergen** | **R/N** | **Exposure** | **Biochemical name/Allergen family** | **Source** |
| --- | --- | --- | --- | --- | --- | --- |
| 38 | *Dermatophagoides*  *pteronyssinus* | Der p 1 | **N** | inh | Cysteine protease | Citeq, Groningen, Netherlands |
| 39 |  | Der p 2 | **N** | inh | NPC2 family | Inbio, Chester, United Kingdom |
| 40 |  | Der p 4 | R | inh | Alpha-amylase | Own-production |
| 41 |  | Der p 5 | R | inh | Group 5 mite allergens | Own-production |
| 42 |  | Der p 7 | R | inh | Bactericidal permeability-increasing like protein | Own-production |
| 43 |  | Der p 10 | R | inh | Tropomyosin | Own-production |
| 44 |  | Der p 18 | R | inh | Chitin-binding domain | Own-production |
| 45 |  | Der p 21 | R | inh | Group 21 mite allergens | Own-production |
| 46 |  | Der p 23 | R | inh | Peritrophin-like protein domain | Own-production |
| 47 |  | Der p 37 | R | inh | Peritrophin-A domain containing protein | Own-production |
| 48 | *Blomia tropicalis* | Blo t 1 | R | inh | Cysteine protease | Own-production |
| 49 |  | Blo t 2 | R | inh | ML-domain protein | Own-production |
| 50 |  | Blo t 5 | R | inh | Group 5 mite allergens | Own-production |
| 51 |  | Blo t 8 | R | inh | Glutathione S-transferase | Own-production |
| 52 |  | Blo t 10 | R | inh | Tropomyosin | Own-production |
| 53 |  | Blo t 12 | R | inh | Group 12 mite allergens | Own-production |
| 54 |  | Blo t 13 | R | inh | Fatty acid-binding protein | Own-production |
| 55 |  | Blo t 21 | R | inh | Group 21 mite allergens | Own-production |
| 56 | *Cockroach* | Bla g 1 | R | inh | Cockroach group 1 (microvilli-like protein) | Own-production |
| 57 |  | Bla g 2 | R | inh | Inactive aspartic protease | Own-production |
| 58 |  | Bla g 5 | R | inh | Glutathione S-transferase | Own-production |
| 59 |  | Bla g 7 | R | inh | Tropomyosin | Own-production |
| 60 | *Cat* | Fel d 1 | R | inh | Uteroglobin | Own-production |
| 61 |  | Fel d 2 | **N** | inh | Serum Albumin | Sigma-Aldrich, Missouri, USA |
| 62 |  | Fel d 3 | R | inh | Cystatin-A | Own-production |
| 63 |  | Fel d 4 | R | inh | Lipocalin | Own-production |
| 64 | *Dog* | Can f 1 | R | inh | Lipocalin | Own-production |
| 65 |  | Can f 2 | R | inh | Lipocalin | Own-production |
| 66 |  | Can f 3 | **N** | inh | Serum Albumin | Sigma-Aldrich, Missouri, USA |
| 67 |  | Can f 4 | R | inh | Lipocalin (Odorant-binding protein) | Own-production |
| 68 |  | Can f 6 | R | inh | Lipocalin | Own-production |
| 69 | *Horse* | Equ c 1 | R | inh | Lipocalin | Own-production |
| 70 |  | Equ c 3 | **N** | inh | Serum Albumin | Sigma-Aldrich, Missouri, USA |
| 71 | *Mouse* | Mus m 1 | R | inh | Lipocalin | Own-production |

**Table S1.** (Continued)

| **No.** | **Allergen-source** | **Allergen** | **R/N** | **Exposure** | **Biochemical name/Allergen family** | **Source** |
| --- | --- | --- | --- | --- | --- | --- |
| 72 | *Peanut* | Ara h 1 | R | f | Storage protein, 7S globulin (Vicilins) | Own-production |
| 73 |  | Ara h 2 | R | f | Storage protein, 2S albumin | Own-production |
| 74 |  | Ara h 3 | R | f | Storage protein, 11S globulin (Legumins) | Own-production |
| 75 |  | Ara h 6 | R | f | Storage protein, 2S albumin | Own-production |
| 76 |  | Ara h 8 | R | f | PR-10 | Own-production |
| 77 |  | Ara h 9 | R | f | nsLTP, type 1 | Own-production |
| 78 | *Hazelnut* | Cor a 1.0401 | R | f | PR-10 | Own-production |
| 79 | *Soy* | Gly m 4 | R | f | PR-10 | Own-production |
| 80 |  | Gly m 5 | **N** | f | Storage protein, 7S globulin (Vicilins) | Inbio, Chester, United Kingdom |
| 81 |  | Gly m 6 | **N** | f | Storage protein, 11S globulin (Legumins) | Inbio, Chester, United Kingdom |
| 82 | *Wheat* | Tri a 20 | R | f | Gamma gliadin | Own-production |
| 83 |  | Tri a 37 | R | f | Alpha purothionin | Own-production |
| 84 | *Apple* | Mal d 1 | R | f | PR-10 | Own-production |
| 85 | *Peach* | Pru p 1 | R | f | PR-10 | Own-production |
| 86 |  | Pru p 3 | R | f | nsLTP, type 1 | Own-production |
| 87 | *Cow’s Milk* | Bos d 4 | **N** | f | Alpha-lactalbumin | Sigma-Aldrich, Missouri, USA |
| 88 |  | Bos d 5 | **N** | f | Beta-lactoglobulin | Sigma-Aldrich, Missouri, USA |
| 89 |  | Bos d 6 | **N** | f, inh | Serum Albumin | Thermofisher, Massachusetts, USA |
| 90 |  | Bos d 8 | **N** | f | Caseins | Sigma-Aldrich, Missouri, USA |
| 91 |  | Bos d Lf | **N** | f | Transferrin | Sigma-Aldrich, Missouri, USA |
| 92 | *Egg* | Gal d 1 | **N** | f | Ovomucoid | Sigma-Aldrich, Missouri, USA |
| 93 |  | Gal d 2 | **N** | f | Ovalbumin | Sigma-Aldrich, Missouri, USA |
| 94 |  | Gal d 3 | **N** | f | Ovotransferrin (Conalbumin) | Sigma-Aldrich, Missouri, USA |
| 95 |  | Gal d 4 | **N** | f | Lysozyme C | Sigma-Aldrich, Missouri, USA |
| 96 | *Codfish* | Gad c 1 | R | f | Parvalbumin | Own-production |
| 97 | *Shrimp* | Pen m 1 | R | f | Tropomyosin | Own-production |
| 98 |  | Pen m 2 | R | f | Arginine Kinase-like protein | Own-production |
| 99 | *Snail* | Hel as 1 | R | f | Tropomyosin | Own-production |
| 100 | *Anisakis* | Ani s 3 | R | f | Tropomyosin | Own-production |
| 101 | *Bee* | Api m 1 | R | syst | Insect venom, Phospholipase A2 | Own-production |
| 102 |  | Api m 2 | R | syst | Insect venom, Hyaluronidase | Own-production |
| 103 | *Wasp* | Ves v 1 | R | syst | Insect venom, Phospholipase A1 | Own-production |
| 104 |  | Ves v 2 | R | syst | Insect venom, Hyaluronidase | Own-production |
| 105 |  | Ves v 5 | R | syst | Insect venom, Antigen 5 | Own-production |
| 106 | *Horseradish* | HRP | N | / | CCD-marker | Sigma-Aldrich, Missouri, USA |

**Table S2.** Frequencies of IgE recognition and median IgE levels to respiratory allergens among patients with respiratory allergy in the Algiers study population (N=417). For sensitization frequencies, *p* values were obtained using logistic regression adjusted for sex and the interaction between sex and age. For specific IgE levels, *p* values were obtained using ANOVA adjusted for sex and the interaction between sex and age.

|  | | **Patients**  **(N=417)** | **Children**  **(N=173)** | **Adults**  **(N=244)** | ***p*** |
| --- | --- | --- | --- | --- | --- |
| **Der p 1** | - n (%) - Median [Q1-Q3] | 243 (58.3)  20.5 [8.8-40.2] | 135 (78)  22.9 [11.4-49.1] | 108 (44.3)  16.7 [5.9-29.2] | **˂0.001**  **0.003** |
| **Der p 2** | - n (%) - Median [Q1-Q3] | 238 (57.1)  28 [11.4-55.3] | 125 (72.3)  34.4 [12-67.5] | 113 (46.3)  25 [9.6-46.1] | **˂0.001**  0.128 |
| **Der p 4** | - n (%) - Median [Q1-Q3] | 37 (8.9)  0.6 [0.4-2.5] | 11 (6.4)  0.5 [0.3-3.1] | 26 (10.7)  0.8 [0.5-2.2] | 0.136  0.569 |
| **Der p 5** | - n (%) - Median [Q1-Q3] | 137 (32.9)  24.1 [7.9-59.8] | 77 (44.5)  36.2 [14-80.8] | 60 (24.6)  11.3 [3.6-37.2] | **˂0.001**  **˂0.001** |
| **Der p 7** | - n (%) - Median [Q1-Q3] | 88 (21.1)  8.3 [3.2-17.1] | 50 (28.9)  11.2 [5.7-25.5] | 38 (15.6)  3.8 [1.7-11.9] | **0.002**  **˂0.001** |
| **Der p 10** | - n (%) - Median [Q1-Q3] | 62 (14.9)  11.4 [3.2-48.3] | 29 (16.8)  16.7 [7.1-73.5] | 33 (13.5)  6 [1.4-24.8] | 0.349  **0.010** |
| **Der p 18** | - n (%) - Median [Q1-Q3] | 16 (3.8)  1.2 [0.6-3.3] | 14 (8.1)  1.4 [0.8-3.3] | 2 (0.8)  0.6 [0.6-0.6] | **˂0.001**  n.a |
| **Der p 21** | - n (%) - Median [Q1-Q3] | 131 (31.4)  12 [3.5-39.8] | 70 (40.5)  24.8 [8.6-54.6] | 61 (25)  7.2 [2-18.2] | **0.001**  **˂0.001** |
| **Der p 23** | - n (%) - Median [Q1-Q3] | 271 (65)  31 [12.2-64.7] | 128 (74)  49.7 [23.1-86.4] | 143 (58.6)  20.2 [6.5-44.6] | **0.001**  **˂0.001** |
| **Der p37** | - n (%) - Median [Q1-Q3] | 37 (8.9)  1.7 [0.7-8.7] | 18 (10.4)  4.5 [0.8-16.4] | 19 (7.8)  1.3 [0.7-5.6] | 0.347  0.203 |
| **Blo t 2** | - n (%) - Median [Q1-Q3] | 4 (1)  0.4 [0.4-0.4] | 4 (2.3)  0.4 [0.4-0.4] | 0 (0)  / | n.a  n.a |
| **Blo t 5** | - n (%) - Median [Q1-Q3] | 94 (22.5)  4.7 [1.2-23.6] | 42 (24.3)  5 [1.7-25.5] | 52 (21.3)  3.8 [0.9-18.1] | 0.563  0.475 |
| **Blo t 8** | - n (%) - Median [Q1-Q3] | 1 (0.2)  0.4 [0.4-0.4] | 1 (0.6)  0.4 [0.4-0.4] | 0 (0)  / | n.a  n.a |
| **Blo t 10** | - n (%) - Median [Q1-Q3] | 57 (13.7)  7 [2.7-30.1] | 29 (16.8)  8.9 [4-41.2] | 28 (11.5)  5.7 [1.7-27.7] | 0.121  0.237 |
| **Blo t 12** | - n (%) - Median [Q1-Q3] | 4 (1)  0.4 [0.3-0.7] | 3 (1.7)  0.5 [0.4-0.9] | 1 (0.4)  0.3 [0.3-0.3] | 0.312  n.a |
| **Blo t 13** | - n (%) - Median [Q1-Q3] | 4 (1)  3.8 [1.2-6.2] | 3 (1.7)  6.1 [3.2-6.2] | 1 (0.4)  1.5 [1.5-1.5] | 0.312  n.a |
| **Blo t 21** | - n (%) - Median [Q1-Q3] | 98 (23.5)  6.7 [1.7-25] | 38 (22)  8.4 [1-26.4] | 60 (24.6)  5.9 [2-21.9] | 0.506  0.597 |
| **Bla g 5** | - n (%) - Median [Q1-Q3] | 3 (0.7)  0.9 [0.6-3.2] | 1 (0.6)  5.4 [5.4-5.4] | 2 (0.8)  0.6 [0.3-0.9] | 1  n.a |
| **Bla g 7** | - n (%) - Median [Q1-Q3] | 69 (16.5)  13.2 [2.5-69.6] | 32 (18.5)  19.7 [6.2-157.9] | 37 (15.2)  6.6 [1.1-47.9] | 0.385  **0.014** |
| **Alt a 1** | - n (%) - Median [Q1-Q3] | 11 (2.6)  1.9 [1.7-4.8] | 7 (4)  1.9 [1.7-3.7] | 4 (1.6)  3.3 [1.5-4.9] | 0.213  n.a |
| **Asp f 3** | - n (%) - Median [Q1-Q3] | 12 (2.9)  0.6 [0.4-2.6] | 2 (1.2)  7.1 [5.9-8.3] | 10 (4.1)  0.5 [0.4-0.9] | 0.134  n.a |
| **Fel d 1** | - n (%) - Median [Q1-Q3] | 90 (21.6)  4.8 [2.3-14.4] | 43 (24.9)  8.2 [2.4-20.7] | 47 (19.3)  4.2 [1.9-10.7] | 0.123  0.314 |
| **Fel d 2** | - n (%) - Median [Q1-Q3] | 4 (1)  6.6 [4.8-13.7] | 3 (1.7)  7.2 [6.6-20.3] | 1 (0.4)  1.5 [1.5-1.5] | 0.312  n.a |
| **Fel d 3** | - n (%) - Median [Q1-Q3] | 4 (1)  1.3 [0.6-3.1] | 2 (1.2)  4.4 [1.9-6.8] | 2 (0.8)  0.5 [0.3-0.7] | 1  n.a |
| **Fel d 4** | - n (%) - Median [Q1-Q3] | 6 (1.4)  1.3 [0.5-8.9] | 1 (0.6)  0.4 [0.4-0.4] | 5 (2)  1.9 [0.7-11.3] | 0.408  n.a |
| **Can f 1** | - n (%) - Median [Q1-Q3] | 4 (1)  3.7 [0.8-9.3] | 2 (1.2)  0.8 [0.7-0.8] | 2 (0.8)  11.9 [6.7-17.1] | 1  n.a |
| **Can f 2** | - n (%) - Median [Q1-Q3] | 1 (0.2)  0.5 [0.5-0.5] | 0 (0)  / | 1 (0.4)  0.5 [0.5-0.5] | n.a  n.a |
| **Can f 3** | - n (%) - Median [Q1-Q3] | 5 (1.2)  1.7 [0.4-5.6] | 4 (2.3)  3.6 [1.3-7.7] | 1 (0.4)  0.4 [0.4-0.4] | 0.165  n.a |
| **Can f 4** | - n (%) - Median [Q1-Q3] | 1 (0.2)  21.1 [21.1 -21.1] | 0 (0)  / | 1 (0.4)  21.1 [21.1 -21.1] | n.a  n.a |
| **Mus m 1** | - n (%) - Median [Q1-Q3] | 4 (1)  2.2 [0.8-5.1] | 1 (0.6)  9.4 [9.4-9.4] | 3 (1.2)  0.8 [0.8-2.2] | 0.645  n.a |
| **Equ c 1** | - n (%) - Median [Q1-Q3] | 4 (1)  1.4 [0.9-2.3] | 1 (0.6)  0.9 [0.9-0.9] | 3 (1.2)  2 [1.4-2.6] | 0.645  n.a |
| **Equ c 3** | - n (%) - Median [Q1-Q3] | 3 (0.7)  1.4 [0.9-9.3] | 3 (1.7)  1.4 [0.9-9.3] | 0 (0)  / | n.a  n.a |
| **Ole e 1** | - n (%) - Median [Q1-Q3] | 100 (24)  4.2 [1.1-12] | 32 (18.5)  3.7 [1.1-12.1] | 68 (27.9)  4.1 [1.4-11.7] | **0.037**  0.564 |
| **Ole e 2** | - n (%) - Median [Q1-Q3] | 1 (0.2)  0.3 [0.3-0.3] | 1 (0.6)  0.3 [0.3-0.3] | 0 (0)  / | n.a  n.a |
| **Ole e 3** | - n (%) - Median [Q1-Q3] | 10 (2.4)  2.7 [1.5-7.6] | 5 (2.9)  2.5 [1.8-11] | 5 (2)  2.9 [1.4-4.4] | 0.839  0.084 |
| **Ole e 5** | - n (%) - Median [Q1-Q3] | 15 (3.6)  1.5 [0.5-3.8] | 8 (4.6)  1.8 [1.1-3.7] | 7 (2.9)  0.9 [0.5-4.7] | 0.343  0.877 |
| **Ole e 6** | - n (%) - Median [Q1-Q3] | 1 (0.2)  0.3 [0.3-0.3] | 1 (0.6)  0.3 [0.3-0.3] | 0 (0)  / | n.a  n.a |
| **Ole e 7** | - n (%) - Median [Q1-Q3] | 1 (0.2)  0.4 [0.4-0.4] | 0 (0)  / | 1 (0.4)  0.4 [0.4-0.4] | n.a  n.a |
| **Ole e 8** | - n (%) - Median [Q1-Q3] | 2 (0.5)  0.5 [0.4-0.6] | 2 (1.2)  0.5 [0.4-0.6] | 0 (0)  / | n.a  n.a |
| **Ole e 9** | - n (%) - Median [Q1-Q3] | 1 (0.2)  0.3 [0.3-0.3] | 0 (0)  / | 1 (0.4)  0.3 [0.3-0.3] | n.a  n.a |
| **Ole e 10** | - n (%) - Median [Q1-Q3] | 1 (0.2)  9.2 [9.2-9.2] | 0 (0)  / | 1 (0.4)  9.2 [9.2-9.2] | n.a  n.a |
| **Bet v 2** | - n (%) - Median [Q1-Q3] | 1 (0.2)  0.3 [0.3-0.3] | 1 (0.6)  0.3 [0.3-0.3] | 0 (0)  / | n.a  n.a |
| **Bet v 4** | - n (%) - Median [Q1-Q3] | 10 (2.4)  1.8 [0.8-5.4] | 4 (2.3)  4.8 [1.1-11.1] | 6 (2.5)  1.7 [0.6-4] | n.a  n.a |
| **Pla a 1** | - n (%) - Median [Q1-Q3] | 1 (0.2)  0.3 [0.3-0.3] | 1 (0.6)  0.3 [0.3-0.3] | 0 (0)  / | n.a  n.a |
| **Par j 2** | - n (%) - Median [Q1-Q3] | 33 (7.9)  8.7 [2.6-26.6] | 9 (5.2)  6.9 [1.5-8.3] | 24 (9.8)  12.8 [2.9-35.1] | 0.110  0.672 |
| **Amb a 1** | - n (%) - Median [Q1-Q3] | 7 (1.7)  0.6 [0.5-1.4] | 0 (0)  / | 7 (2.9)  0.6 [0.5-1.4] | n.a  n.a |
| **Amb a 4** | - n (%) - Median [Q1-Q3] | 14 (3.4)  1.1 [0.6-1.9] | 4 (2.3)  0.5 [0.4-1.1] | 10 (4.1)  1.2 [0.8-2.7] | 0.413  n.a |
| **Art v 1** | - n (%) - Median [Q1-Q3] | 8 (1.9)  2.9 [2.5-8] | 2 (1.2)  1.8 [0.8-2.8] | 6 (2.5)  4.8 [2.9-10.5] | 0.478  n.a |
| **Art v 3** | - n (%) - Median [Q1-Q3] | 3 (0.7)  0.4 [0.3-1.5] | 1 (0.6)  1.5 [1.5-1.5] | 2 (0.8)  0.3 [0.3-0.3] | 1  n.a |
| **Pla l 1** | - n (%) - Median [Q1-Q3] | 1 (0.2)  0.3 [0.3-0.3] | 1 (0.6)  0.3 [0.3-0.3] | 0 (0)  / | n.a  n.a |
| **Phl p 1** | - n (%) - Median [Q1-Q3] | 17 (4.1)  1 [0.5-2] | 3 (1.7)  0.6 [0.4-1] | 14 (5.7)  1.5 [0.5-2.8] | **0.046**  n.a |
| **Phl p 2** | - n (%) - Median [Q1-Q3] | 11 (2.6)  7.6 [1.4-12.6] | 0 (0)  / | 11 (4.5)  7.6 [1.4-12.6] | n.a  n.a |
| **Phl p 3** | - n (%) - Median [Q1-Q3] | 13 (3.1)  7.3 [1.4-31.3] | 0 (0)  / | 13 (5.3)  7.3 [1.4-31.3] | n.a  n.a |
| **Phl p 5b** | - n (%) - Median [Q1-Q3] | 6 (1.4)  9.1 [6-9.7] | 1 (0.6)  9.7 [9.7-9.7] | 5 (2)  8.9 [4.4-15.5] | 0.408  n.a |
| **Phl p 6** | - n (%) - Median [Q1-Q3] | 7 (1.7)  1.4 [1-3.1] | 3 (1.7)  1.1 [1-1.4] | 4 (1.6)  2.8 [1.5-3.5] | 1  n.a |
| **Phl p 7** | - n (%) - Median [Q1-Q3] | 8 (1.9)  3.8 [2-8.8] | 4 (2.3)  6.6 [2.2-19.3] | 4 (1.6)  3.5 [1.9-6] | 0.723  n.a |
| **Phl p 12** | - n (%) - Median [Q1-Q3] | 1 (0.2)  0.5 [0.5-0.5] | 1 (0.6)  0.5 [0.5-0.5] | 0 (0)  / | n.a  n.a |
| **HRP (CCD)** | - n (%) - Median [Q1-Q3] | 42 (10.1)  1.3 [0.6-4.9] | 10 (5.8)  0.9 [0.6-10.4] | 32 (13.1)  1.5 [0.7-4.7] | **0.014**  0.965 |

**Table S3.** Mono- and co-sensitizations to allergens belonging to the same protein family (analyzed among the Algerian study population (N=539)).

| **Tropomyosins** (84 reactive patients) | | | | | | | | | **Lipocalins** (14 reactive patients) | | | | | | | |
| --- | --- | --- | --- | --- | --- | --- | --- | --- | --- | --- | --- | --- | --- | --- | --- | --- |
| Nbr of profiles | **Patients** | | **Allergens** | | | | | | **Patients** | | **Allergens** | | | | | |
|  | n | % | **Ani s 3** | **Bla g 7** | **Blo t 10** | **Der p 10** | **Hel as 1** | **Pen m 1** | n | % | **Can f 1** | **Can f 2** | **Can f 4** | **Equ c 1** | **Fel d 4** | **Mus m 1** |
| 1 | 54 | 64.29 | **+** | **+** | **+** | **+** | **+** | **+** | 4 | 28.57 | **-** | **-** | **-** | **-** | **+** | **-** |
| 2 | 14 | 16.67 | - | **+** | - | - | - | - | 2 | 14.29 | **+** | **-** | **-** | **-** | **-** | **-** |
| 3 | 4 | 4.76 | **+** | **+** | **+** | **+** | - | **+** | 2 | 14.29 | **-** | **+** | **-** | **-** | **-** | **-** |
| 4 | 4 | 4.76 | - | **+** | - | **+** | - | **+** | 1 | 7.14 | **-** | **-** | **-** | **+** | **-** | **-** |
| 5 | 2 | 2.38 | - | **+** | - | **+** | - | - | 1 | 7.14 | **-** | **-** | **-** | **-** | **-** | **+** |
| 6 | 2 | 2.38 | - | **+** | **+** | **+** | - | **+** | 1 | 7.14 | **+** | **-** | **-** | **+** | **-** | **-** |
| 7 | 2 | 2.38 | - | - | - | - | **+** | - | 1 | 7.14 | **+** | **-** | **-** | **-** | **-** | **+** |
| 8 | 1 | 1.19 | - | - | - | **+** | - | - | 1 | 7.14 | **-** | **-** | **-** | **+** | **+** | **+** |
| 9 | 1 | 1.19 | - | **+** | **+** | **+** | **+** | **+** | 1 | 7.14 | **+** | **-** | **+** | **-** | **+** | **+** |

| **nsLTP** (58 reactive patients) | | | | | | | | | **Seed storage proteins** (26 reactive patients) | | | | | | | **Serum albumins** (44 reactive patients) | | | | | |
| --- | --- | --- | --- | --- | --- | --- | --- | --- | --- | --- | --- | --- | --- | --- | --- | --- | --- | --- | --- | --- | --- |
| Nbr of profiles | **Patients** | | **Allergens** | | | | | | **Patients** | | **Allergens** | | | | | **Patients** | | **Allergens** | | | |
|  | n | % | **Ara h 9** | **Art v 3** | **Ole e 7** | | **Par j 2** | **Pru p 3** | n | % | **Ara h 2** | **Ara h 6** | **Ara h 1** | **Gly m 5** | **Gly m 6** | n | % | **Bos d 6** | **Can f 3** | **Equ c 3** | **Fel d 2** |
| 1 | 33 | 56.90 | **-** | **-** | **-** | **+** | | **-** | 5 | 19.23 | **+** | **-** | **-** | **-** | **-** | 18 | 40.9 | **+** | **-** | **-** | **-** |
| 2 | 8 | 13.79 | **+** | **-** | **-** | **-** | | **-** | 4 | 15.38 | **-** | **+** | **-** | **-** | **-** | 11 | 25 | **+** | **+** | **+** | **+** |
| 3 | 5 | 8.62 | **-** | **+** | **-** | **+** | | **-** | 4 | 15.38 | **-** | **-** | **+** | **-** | **-** | 7 | 15.9 | **+** | **+** | **-** | **-** |
| 4 | 4 | 6.90 | **-** | **+** | **-** | **-** | | **-** | 3 | 11.54 | **-** | **-** | **-** | **+** | **-** | 3 | 6.82 | **+** | **+** | **-** | **+** |
| 5 | 3 | 5.17 | **+** | **+** | **-** | **-** | | **+** | 3 | 11.54 | **+** | **+** | **-** | **-** | **-** | 2 | 4.55 | **+** | **+** | **+** | **-** |
| 6 | 1 | 1.72 | **-** | **+** | **-** | **+** | | **+** | 2 | 7.69 | **-** | **-** | **+** | **+** | **-** | 1 | 2.27 | **-** | **+** | **-** | **+** |
| 7 | 1 | 1.72 | **+** | **-** | **-** | **-** | | **+** | 2 | 7.69 | **+** | **+** | **+** | **-** | **-** | 1 | 2.27 | **+** | **-** | **+** | **+** |
| 8 | 1 | 1.72 | **-** | **+** | **-** | **-** | | **+** | 1 | 3.85 | **-** | **-** | **+** | **+** | **+** | 1 | 2.27 | **-** | **+** | **-** | **-** |
| 9 | 1 | 1.72 | **-** | **-** | **+** | **-** | | **-** | 1 | 3.85 | **+** | **-** | **+** | **-** | **-** |  |  |  |  |  |  |
| 10 | 1 | 1.72 | **-** | **-** | **-** | **-** | | **+** | 1 | 3.85 | **-** | **-** | **-** | **-** | **+** |  |  |  |  |  |  |

| **2 EF-hands calcium binding proteins** (11 reactive patients) | | | | | | **Profilins** (05 reactive patients) | | | | | | | **PR10** (13 reactive patients) | | | | |
| --- | --- | --- | --- | --- | --- | --- | --- | --- | --- | --- | --- | --- | --- | --- | --- | --- | --- |
| Nbr of profiles | **Patients** | | **Allergens** | | | **Patients** | | **Allergens** | | | | | **Patients** | | **Allergens** | | |
|  | n | % | **Bet v 4** | **Ole e 3** | **Phl p 7** | n | % | **Amb a 8** | **Bet v 2** | **Hev b 8** | **Ole e 2** | **Phl p 12** | n | % | **Cor a 1.04** | **Mal d 1** | **Pru p 1** |
| 1 | 8 | 72.73 | **+** | **+** | **+** | 2 | 40 | **-** | **+** | **+** | **+** | **+** | 11 | 84.62 | **+** | **-** | **-** |
| 2 | 1 | 9.09 | **+** | **+** | **-** | 1 | 20 | **+** | **+** | **+** | **+** | **+** | 1 | 7.69 | **-** | **-** | **+** |
| 3 | 1 | 9.09 | **+** | **-** | **-** | 1 | 20 | **-** | **-** | **-** | **+** | **+** | 1 | 7.69 | **-** | **+** | **+** |
| 4 | 1 | 9.09 | **-** | **+** | **-** | 1 | 20 | **-** | **-** | **-** | **+** | **-** |  |  |  |  |  |

**Table S4.** Frequencies of IgE recognition and median IgE levels to respiratory allergens among Turkish (Istanbul and Samsun) population (N=272). For sensitization frequencies, *p* values were obtained using logistic regression adjusted for sex and the interaction between sex and age. For specific IgE levels, *p* values were obtained using analysis of variance adjusted for sex and the interaction between sex and age.

|  |  | **Patients**  **(N=272)** | **Children**  **(N=133)** | **Adults**  **(N=139)** | ***p*** |
| --- | --- | --- | --- | --- | --- |
| **Der p 1** | - n (%) - Median [Q1-Q3] | 154 (56.6)  16.7 [4.1-47.1] | 86 (64.7)  27.5 [7.9-78.7] | 68 (48.9)  7.7 [3.2-22.6] | **0.018**  **˂0.001** |
| **Der p 2** | - n (%) - Median [Q1-Q3] | 169 (62.1)  7.8 [2.6-24.4] | 92 (69.2)  5.6 [1.7-11.0] | 77 (55.4)  16.2 [3.5-39.9] | **0.019**  **0.007** |
| **Der p 5** | - n (%) - Median [Q1-Q3] | 57 (21.0)  10.8 [2.4-24.6] | 28 (21.1)  15.9 [4.4-35.9] | 29 (20.9)  5.0 [2.2-17.8] | 0.704  0.372 |
| **Der p 7** | - n (%) - Median [Q1-Q3] | 55 (20.2)  9.7 [3.6-19.7] | 31 (23.3)  16.2 [8.6-31.2] | 24 (17.3)  4.9 [1.0-10.9] | 0.187  **0.033** |
| **Der p 21** | - n (%) - Median [Q1-Q3] | 54 (19.9)  9.8 [2.8-29.0] | 23 (17.3)  28.5 [9.2-75.5] | 31 (22.3)  5.0 [2.4-14.8] | 0.301  **<0.001** |
| **Der p 23** | - n (%) - Median [Q1-Q3] | 169 (62.1)  12.8 [4.0-35.2] | 81 (60.9)  15.1 [7.8-34.7] | 88 (63.3)  9.4 [2.6-37.6] | 0.581  0.443 |
| **Der p 37** | - n (%) - Median [Q1-Q3] | 25 (9.2)  1.4 [0.6-7.8] | 9 (6.8)  1.0 [0.5-35.8] | 16 (11.5)  2.5 [0.6-7.1] | 0.201  0.086 |
| **Phl p 1** | - n (%) - Median [Q1-Q3] | 80 (29.4)  4.1 [0.7-22.8] | 42 (31.6)  8.6 [0.5-44.4] | 38 (27.3)  2.9 [0.8-12.2] | 0.910  **0.028** |
| **Phl p 2** | - n (%) - Median [Q1-Q3] | 11 (4.0)  3.2 [1.2-8.7] | 2 (1.5)  7.0 [2.7-n.a] | 9 (6.5)  3.2 [0.8-8.4] | 0.061  n.a |
| **Phl p 3** | - n (%) - Median [Q1-Q3] | 17 (6.3)  5.2 [0.5-22.4] | 5 (3.8)  0.3 [0.3-16.9] | 12 (8.6)  8.5 [1.6-25.8] | 0.122  0.414 |
| **Phl p 5** | - n (%) - Median [Q1-Q3] | 20 (7.4)  5.9 [1.2-32.7] | 13 (9.8)  12.3 [2.2-40.9] | 7 (5.0)  2.9 [0.5-9.2] | 0.878  0.953 |
| **Phl p 6** | - n (%) - Median [Q1-Q3] | 18 (6.6)  2.4 [0.8-7.6] | 12 (9.0)  2.4 [0.8-5.4] | 6 (4.3)  4.7 [0.6-9.5] | 0.763  0.958 |
| **Fel d 1** | - n (%) - Median [Q1-Q3] | 62 (22.8)  4.0 [1.2-30.3] | 31 (23.3)  2.5 [1.1-10.9] | 31 (22.3)  8.4 [1.8-47.1] | 0.773  0.288 |
| **Fel d 2** | - n (%) - Median [Q1-Q3] | 22 (8.1)  1.1 [0.6-2.1] | 4 (3.0)  0.5 [0.3-3.2] | 18 (12.9)  1.1 [0.6-2.1] | **0.003**  n.a |
| **Fel d 4** | - n (%) - Median [Q1-Q3] | 16 (5.9)  4.5 [1.0-11.1] | 8 (6.0)  5.3 [2.1-11.1] | 8 (5.8)  3.7 [0.8-12.1] | 0.281  0.499 |
| **Amb a 1** | - n (%) - Median [Q1-Q3] | 52 (19.1)  7.8 [1.9-21.9] | 16 (12.0)  6.6 [1.0-11.9] | 36 (25.9)  10.6 [2.1-27.9] | **0.002**  0.176 |
| **Amb a 8** | - n (%) - Median [Q1-Q3] | 28 (10.3)  0.7 [0.3-1.0] | 4 (3.0)  4.2 [1.2-17.0] | 24 (17.3)  0.5 [0.3-0.9] | **˂0.001**  n.a |
| **Ole e 1** | - n (%) - Median [Q1-Q3] | 19 (6.9)  2.1 [0.8-7.4] | 4 (3.0)  0.9 [0.6-9.9] | 15 (10.7)  2.6 [0.8-7.4] | **0.016**  n.a |
| **Art v 1** | - n (%) - Median [Q1-Q3] | 9 (3.3)  1.4 [0.8-2.9] | 4 (3.0)  1.3 [0.8-8.0] | 5 (3.5)  1.4 [0.5-2.8] | 1  n.a |

**Table S5.** Frequencies of IgE recognition and median IgE levels to food allergens among patients from Algiers with food allergies (N=137). For sensitization frequencies, *p* values were obtained using logistic regression adjusted for sex and the interaction between sex and age. For specific IgE levels, *p* values were obtained using analysis of variance adjusted for sex and the interaction between sex and age.

|  | | | **Patients**  **(N=137)** | **Children**  **(N=97)** | **Adults**  **(N=40)** | ***p*** |
| --- | --- | --- | --- | --- | --- | --- |
| **Bos d 4** | - n (%) - Median [Q1-Q3] | | 25 (18.2)  1.1 [0.6-1.9] | 25 (25.8)  1.1 [0.6-1.9] | 0 (0)  / | n.a  n.a |
| **Bos d 5** | - n (%) - Median [Q1-Q3] | | 26 (19)  1.2 [0.6-2.2] | 26 (26.8)  1.2 [0.6-2.2] | 0 (0)  / | n.a  n.a |
| **Bos Lf** | - n (%) - Median [Q1-Q3] | | 5 (3.6)  3.3 [1-5.6] | 5 (5.2)  3.3 [1-5.6] | 0 (0)  / | n.a  n.a |
| **Bos d 8** | - n (%) - Median [Q1-Q3] | | 24 (17.5)  0.6 [0.4-2.2] | 24 (24.7)  0.6 [0.4-2.2] | 0 (0)  / | n.a  n.a |
| **Bos d 6** | - n (%) - Median [Q1-Q3] | | 38 (27.7)  1.3 [0.7-2.8] | 37 (38.1)  1.3 [0.7-2.8] | 1 (2.5)  2.5 [2.5-2.5] | **˂0.001**  n.a |
| **Gal d 1** | - n (%) - Median [Q1-Q3] | | 11 (8)  0.8 [0.5-1.5] | 11 (11.3)  0.8 [0.5-1.5] | 0 (0)  / | n.a  n.a |
| **Gal d 2** | - n (%) - Median [Q1-Q3] | | 10 (7.3)  0.7 [0.5-1] | 10 (10.3)  0.7 [0.5-1] | 0 (0)  / | n.a  n.a |
| **Gal d 3** | - n (%) - Median [Q1-Q3] | | 8 (5.8)  1.6 [0.9-4] | 8 (8.2)  1.6 [0.9-4] | 0 (0)  / | n.a  n.a |
| **Gal d 4** | - n (%) - Median [Q1-Q3] | | 9 (6.6)  0.8 [0.5-1.8] | 8 (8.2)  0.8 [0.6-1.1] | 1 (2.5)  2.1 [2.1-2.1] | 0.283  n.a |
| **Ara h 1** | - n (%) - Median [Q1-Q3] | | 8 (5.8)  2.2 [0.6-12.6] | 8 (8.2)  2.2 [0.6-12.6] | 0 (0)  / | n.a  n.a |
| **Ara h 2** | - n (%) - Median [Q1-Q3] | | 10 (7.3)  1.4 [0.7-1.9] | 10 (10.3)  1.4 [0.7-1.9] | 0 (0)  / | n.a  n.a |
| **Ara h 6** | - n (%) - Median [Q1-Q3] | | 7 (5.1)  0.6 [0.4-2.9] | 7 (7.2)  0.6 [0.4-2.9] | 0 (0)  / | n.a  n.a |
| **Ara h 9** | - n (%) - Median [Q1-Q3] | | 8 (5.8)  2.3 [0.8-7.5] | 6 (6.2)  1.2 [0.8-2.7] | 2 (5)  6.4 [4.1-8.6] | 1  n.a |
| **Cor a 1.04** | - n (%) - Median [Q1-Q3] | | 8 (5.8)  0.4 [0.3-0.9] | 8 (8.2)  0.4 [0.3-0.9] | 0 (0)  / | n.a  n.a |
| **Gly m 5** | - n (%) - Median [Q1-Q3] | | 5 (3.6)  1.2 [0.4-3.8] | 4 (4.1)  0.8 [0.5-1.8] | 1 (2.5)  4.1 [4.1-4.1] | 1  n.a |
| **Gly m 6** | - n (%) - Median [Q1-Q3] | | 2 (1.5)  0.9 [0.3-1.5] | 2 (2.1)  0.9 [0.3-1.5] | 0 (0)  / | n.a  n.a |
| **Gad c 1** | - n (%) - Median [Q1-Q3] | | 11 (8)  3.3 [1-8.5] | 9 (9.3)  3.3 [0.6-11.8] | 2 (5)  3.3 [1.4-5.3] | 0.509  n.a |
| **Pen m 1** | - n (%) - Median [Q1-Q3] | | 40 (29.2)  9.8 [2.7-41.1] | 19 (19.6)  15.5 [4.1-89.2] | 21 (52.5)  7.1 [1.6-30.4] | **˂0.001**  0.225 |
| **Pen m 2** | - n (%) - Median [Q1-Q3] | | 8 (5.8)  0.8 [0.5-2.6] | 3 (3.1)  0.8 [0.5-7.5] | 5 (12.5)  1.3 [0.5-2.2] | **0.047**  n.a |
| **Pru p 1** | - n (%) - Median [Q1-Q3] | | 1 (0.7)  1.1 [1.1-1.1] | 0 (0)  / | 1 (2.5)  1.1 [1.1-1.1] | n.a  n.a |
| **Pru p 3** | - n (%) - Median [Q1-Q3] | | 6 (4.4)  1.1 [0.8-1.8] | 4 (4.1)  1 [0.8-1.2] | 2 (5)  1.6 [1.2-1.9] | 1  n.a |
| **Tri a 20** | | - n (%) - Median [Q1-Q3] | 1 (0.7)  5.3 [5.3 -5.3] | 1 (1)  5.3 [5.3 -5.3] | 0 (0)  / | n.a  n.a |
| **HRP (CCD)** | | - n (%) - Median [Q1-Q3] | 15 (10.9)  0.9 [0.6-1.5] | 10 (10.3)  1 [0.6-1.5] | 5 (12.5)  0.9 [0.9-1.4] | 0.709  0.540 |

**Table S6.** Frequencies of IgE recognition and median IgE levels to venom allergens among patients from Algiers with hymenoptera venom allergy (N=56). For sensitization frequencies, *p* values were obtained using logistic regression adjusted for sex and the interaction between sex and age. For specific IgE levels, *p* values were obtained using analysis of variance adjusted for sex and the interaction between sex and age.

|  | | **Patients**  **(N=56)** | **Children**  **(N=25)** | **Adults**  **(N=31)** | ***p*** |
| --- | --- | --- | --- | --- | --- |
| **Api m 1** | - n (%) - Median [Q1-Q3] | 25 (44.6)  0.5 [0.4-0.8] | 14 (56)  0.5 [0.5-0.8] | 11 (35.5)  0.5 [0.4-1] | 0.121  0.443 |
| **Api m 2** | - n (%) - Median [Q1-Q3] | 7 (12.5)  0.7 [0.4-1.2] | 4 (16)  1.2 [0.7-1.8] | 3 (9.7)  0.5 [0.4-0.7] | 0.668  n.a |
| **Ves v 5** | - n (%) - Median [Q1-Q3] | 9 (16.1)  0.4 [0.3-0.5] | 3 (12)  0.5 [0.3-1.1] | 6 (19.4)  0.4 [0.3-0.5] | 0.716  n.a |
| **HRP (CCD)** | - n (%) - Median [Q1-Q3] | 35 (62.5)  1.9 [0.7-5.3] | 16 (64)  3 [1.3-8.1] | 19 (61.3)  1.4 [0.5-2.2] | 0.164  0.960 |

**Figure S1.** Comparison of spIgE levels to *Dermatophagoides pteronyssinus* antigens between children and adults in the Algiers study population. *p* value * ˂0.05 ** ˂0.01 *** ˂0.001. *p* values were obtained using analysis of variance adjusted for sex and the interaction between sex and age.

**Figure S2.** Comparison of spIgE levels to outdoor allergens (Ole e 1, Par j 2 and Phl p 1) between children and adults in the Algiers study population.
